# CdiA Effectors Use Modular Receptor-Binding Domains To Recognize Target Bacteria

**DOI:** 10.1128/mBio.00290-17

**Published:** 2017-03-28

**Authors:** Zachary C. Ruhe, Josephine Y. Nguyen, Jing Xiong, Sanna Koskiniemi, Christina M. Beck, Basil R. Perkins, David A. Low, Christopher S. Hayes

**Affiliations:** a Department of Molecular, Cellular and Developmental Biology, University of California, Santa Barbara, Santa Barbara, California, USA; b Biomolecular Science and Engineering Program, University of California, Santa Barbara, Santa Barbara, California, USA; Massachusetts Institute of Technology

**Keywords:** bacterial competition, cell-cell adhesion, self/nonself discrimination, toxin immunity proteins, type V secretion system

## Abstract

Contact-dependent growth inhibition (CDI) systems encode CdiA effectors, which bind to specific receptors on neighboring bacteria and deliver C-terminal toxin domains to suppress target cell growth. Two classes of CdiA effectors that bind distinct cell surface receptors have been identified, but the molecular basis of receptor specificity is not understood. Alignment of BamA-specific CdiA^EC93^ from *Escherichia coli* EC93 and OmpC-specific CdiA^EC536^ from *E. coli* 536 suggests that the receptor-binding domain resides within a central region that varies between the two effectors. In support of this hypothesis, we find that CdiA^EC93^ fragments containing residues Arg1358 to Phe1646 bind specifically to purified BamA. Moreover, chimeric CdiA^EC93^ that carries the corresponding sequence from CdiA^EC536^ is endowed with OmpC-binding activity, demonstrating that this region dictates receptor specificity. A survey of *E. coli* CdiA proteins reveals two additional effector classes, which presumably recognize distinct receptors. Using a genetic approach, we identify the outer membrane nucleoside transporter Tsx as the receptor for a third class of CdiA effectors. Thus, CDI systems exploit multiple outer membrane proteins to identify and engage target cells. These results underscore the modularity of CdiA proteins and suggest that novel effectors can be constructed through genetic recombination to interchange different receptor-binding domains and toxic payloads.

## IMPORTANCE

CdiB/CdiA two-partner secretion proteins mediate interbacterial competition through the delivery of polymorphic toxin domains. This process, known as contact-dependent growth inhibition (CDI), requires stable interactions between the CdiA effector protein and specific receptors on the surface of target bacteria. Here, we localize the receptor-binding domain to the central region of *E. coli* CdiA. Receptor-binding domains vary between CdiA proteins, and *E. coli* strains collectively encode at least four distinct effector classes. Further, we show that receptor specificity can be altered by exchanging receptor-binding regions, demonstrating the modularity of this domain. We propose that novel CdiA effectors are naturally generated through genetic recombination to interchange different receptor-binding domains and toxin payloads.

## INTRODUCTION

Bacteria have long been known to compete with one another using diffusible antibiotics and bacteriocins (1, 2). More recently, type IV, V, and VI secretion systems have been found to mediate interbacterial competition in a proximity-dependent manner (3–6). This phenomenon was first described for *Escherichia coli* isolate EC93, which uses a type Vb secretion system to bind and inhibit the growth of other *E. coli* cells (3). Because this inhibition requires direct cell-to-cell contact, the mechanism has been termed contact-dependent growth inhibition (CDI). Related CDI systems have since been identified and characterized in other Gram-negative bacteria, where they play important roles in interstrain competition and self/nonself discrimination (7–11). CDI is mediated by the CdiB/CdiA family of two-partner secretion proteins. CdiB is an Omp85 transport protein that exports and presents toxic CdiA proteins on the cell surface. CdiA effectors range from 180 to 630 kDa depending on bacterial species (12), but each is predicted to form an elongated filament projecting from the inhibitor cell. Upon binding a specific receptor, CdiA transfers its C-terminal toxin domain (CdiA-CT) into the target bacterium through an incompletely understood translocation pathway (13, 14). CdiA-CT sequences vary considerably between effector proteins, but most toxins either degrade nucleic acids or form pores in target cell membranes (9, 15–19). To prevent self-intoxication, CDI^+^ bacteria produce CdiI immunity proteins, which bind the CdiA-CT domain and neutralize its activity. Because CdiA-CT sequences are highly variable and the domains have distinct three-dimensional structures (16, 20–23), immunity proteins protect against only cognate toxins deployed by sibling cells. Thus, CDI confers a selective advantage against nonisogenic competitors, and toxin immunity polymorphism provides a mechanism for self/nonself discrimination.

CdiA-receptor-binding interactions also play a critical role in self/nonself recognition. Using a genetic approach, Aoki et al. identified BamA as the receptor for the CdiA^EC93^ effector from *E. coli* EC93 (24). BamA is an essential outer membrane β-barrel protein found in all Gram-negative bacteria (25). Though BamA proteins share a high degree of sequence identity between enterobacteria, the surface-exposed extracellular loops vary dramatically between species (26, 27). Thus, CdiA^EC93^ effectively targets *E. coli* strains but does not recognize closely related bacteria like *Enterobacter cloacae*, even though BamA from *E. cloacae* (BamA^ECL^) and BamA from *E. coli* (BamA^Eco^) share ~95% sequence identity (28). Variations in bacterial surface antigens are thought to reflect the selective pressures exerted by bacteriophages and adaptive immune systems (29, 30). Because surface epitopes vary dramatically between bacteria, the CdiA effector family must collectively recognize many distinct receptors. Accordingly, we recently found that CdiA^EC536^ from uropathogenic *E. coli* 536 uses heterotrimeric OmpC-OmpF as a receptor (31). The *E. coli* pan-genome encodes at least 220 distinct OmpC porins, with much of the sequence variability localized to extracellular loops L4 and L5 (31–33). These loops appear to be recognition epitopes for CdiA^EC536^, and consequently, several *E. coli* OmpC variants are not recognized by this effector. Notably, CdiA^EC536^ binds OmpC and OmpF from *E. cloacae* ATCC 13047 (31), suggesting that *E. coli* 536 could use CDI to inhibit other species. Thus, CDI-target cell interactions are complex and idiosyncratic. However, one common and perhaps universal feature is the recognition of “self” receptors, which promotes the autoaggregation of CDI^+^ sibling cells (3, 34). CDI-dependent cell-cell adhesion also contributes significantly to biofilm formation (10, 35–38), and recent work suggests that CDI-mediated toxin exchange is exploited for intercellular communication between siblings (39). These findings indicate that CDI plays an important role in bacterial cooperation, and its toxin delivery activity excludes nonisogenic cells from group activities.

Though CDI recognition epitopes have been localized on BamA and OmpC (28, 31), the corresponding receptor-binding domains in CdiA have not yet been identified. In this report, we use a combination of biochemical and molecular genetic approaches to map the receptor-binding region within *E. coli* CdiA effectors. Previous work with truncated CdiA^EC93^ indicates that its BamA^Eco^-binding domain resides within the N-terminal 2,000 residues (38). We find that CdiA^EC93^ fragments containing residues Arg1358 to Phe1646 interact stably with BamA^Eco^ but not with the closely related BamA^ECL^ protein. These CdiA^EC93^ residues comprise an unannotated central segment located between the FHA-1 and FHA-2 peptide repeat regions. Notably, *E. coli* CdiA proteins can be divided into four major classes based on sequence variation in the putative receptor-binding region. Using chimeric effectors, we show that CdiA^EC93^ is redirected to bind OmpC when residues Ser1347 to Tyr1636 are replaced with the corresponding region from CdiA^EC536^. Given the variability between effector classes, we reasoned that a third class of CdiAs likely recognizes an uncharacterized receptor. Using the CDI^STECO31^ system from *E. coli* STEC_O31 as a model, we identify the outer membrane nucleoside transporter Tsx as the receptor for class III effectors. Thus, CdiA proteins collectively recognize diverse receptors, and their receptor-binding domains are interchangeable between effectors. This modularity suggests that receptor-binding and toxin domains are actively recombined to generate novel effectors.

## RESULTS

### Localization of the BamA-binding region in CdiA^EC93^.

**FIG 1:**
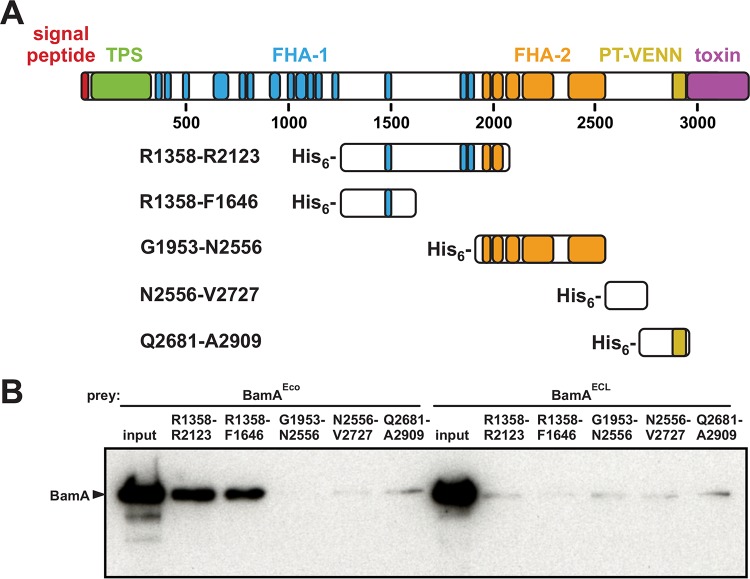
Localization of the BamA^Eco^-binding region within CdiA^EC93^. (A) CdiA^EC93^ domain architecture. Domains and peptide repeat regions are depicted according to InterPro. TPS indicates the two-partner secretion transport domain, and also shown are FHA-1 (PF05594), FHA-2 (PF13332), the pretoxin-VENN domain (PF04829), and the CdiA-CT region. His_6_ epitope-tagged CdiA_EC93_ fragments used for BamA interaction experiments are depicted. (B) Affinity copurification of BamA with His_6_-tagged CdiA^EC93^ fragments. Refolded BamA^Eco^ or BamA^ECL^ was incubated with His_6_-tagged CdiA^EC93^ and then subjected to Ni^2+^-affinity chromatography. The input fraction and final elution samples were analyzed by immunoblotting using polyclonal antisera to BamA^Eco^.

We previously reported that truncated CdiA^EC93^ lacking residues Ala1931 to Lys3242 is exported to the cell surface and retains BamA^Eco^-binding activity (38). Further truncation abrogates adhesin function, but these smaller proteins are not exported efficiently and appear to be degraded rapidly. Therefore, we took a biochemical approach to define the receptor-binding region more precisely. We generated a series of His_6_-tagged CdiA^EC93^ fragments (Fig. 1A) and tested them for binding interactions with purified BamA. Using Ni^2+^ affinity chromatography, we found that BamA^Eco^ copurifies with CdiA^EC93^ fragments containing residues Arg1358 to Phe1646 and Arg1358 to Arg2123 (Fig. 1B). In contrast, CdiA^EC93^ fragments containing the FHA-2 repeat region and the pretoxin-VENN domain did not interact stably with BamA^Eco^ (Fig. 1A and B). To ascertain binding specificity, we also tested for stable interactions with BamA^ECL^ from *E. cloacae*. Though the two BamA proteins share 94.8% sequence identity, BamA^ECL^ did not copurify with any of the His_6_-tagged CdiA^EC93^ fragments (Fig. 1B). Thus, the *in vitro* binding specificity conforms to prior work showing that BamA^ECL^ is not recognized as a receptor by CdiA^EC93^ (28). These data strongly suggest that the BamA^Eco^-binding domain resides between residues Arg1358 and Phe1646 of CdiA^EC93^.

### CdiA receptor-binding domains are modular.

**FIG 2:**
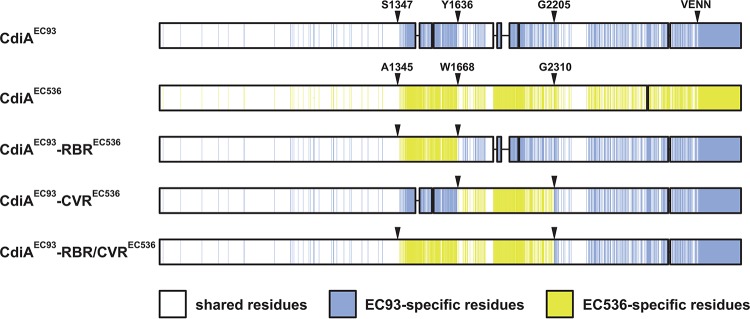
CdiA^EC93^-CdiA^EC536^ chimeras. CdiA^EC93^ and CdiA^EC536^ sequences are shown schematically. Shared residues are indicated in white, and effector-specific residues are indicated in blue and yellow. The residues demarcating the receptor-binding region (RBR) and covarying region (CVR) are indicated, as is the VENN motif that defines the CdiA-CT toxin region.

**FIG 3:**
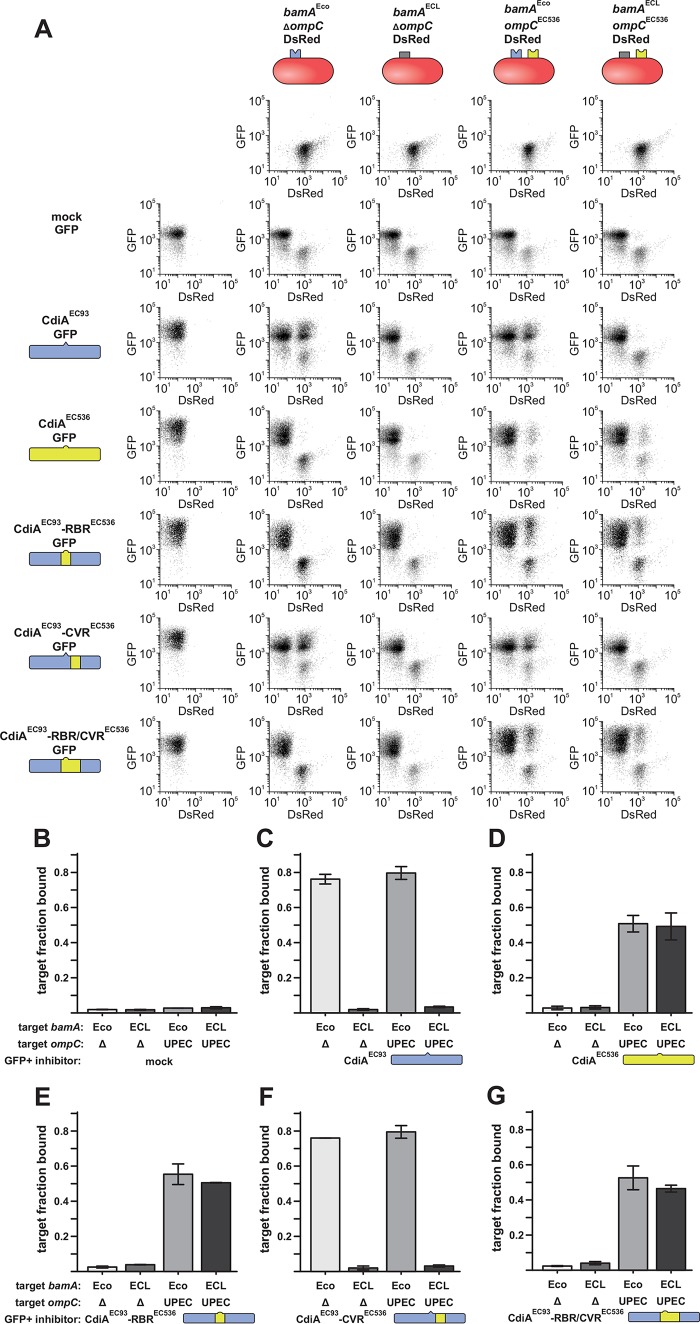
Cell-cell adhesion. (A) Flow cytometric analysis of CDI-dependent cell-cell adhesion. GFP-labeled inhibitor cells were mixed at a 5:1 ratio with DsRed-labeled target bacteria, and the cell suspension was analyzed by flow cytometry for dual green/red fluorescent events. CdiA protein identity is indicated schematically in the left margin, and target cell genetic backgrounds are indicated along the top. (B to G) CdiA-dependent cell-cell adhesion was quantified for each effector protein: mock CDI^−^ (B), CdiA^EC93^ (C), CdiA^EC536^ (D), CdiA^EC93^ with the receptor-binding region (RBR) from CdiA^EC536^ (E), CdiA^EC93^ with the covarying region (CVR) from CdiA^EC536^ (F), and CdiA^EC93^ with both RBR and CVR from CdiA^EC536^ (G). The fraction of target bacteria bound to inhibitor cells was quantified for two independent experiments. Data are presented as averages ± standard errors. UPEC, uropathogenic *E. coli*.

The putative receptor-binding region of CdiA^EC93^ (from Ser1379 to Tyr1636) shares only ~24% sequence identity with the corresponding region of CdiA^EC536^ (Gln1377 to Trp1668) from uropathogenic *E. coli* 536 (Fig. 2; see also Fig. S1 in the supplemental material). Therefore, we reasoned that sequence divergence over this region could determine receptor specificity. To test this hypothesis, we replaced residues Ser1347 to Tyr1636 of CdiA^EC93^ with Ala1345 to Trp1668 from CdiA^EC536^ (Fig. 2) and determined the receptor-binding specificity of the resulting chimera using a flow cytometry-based cell-cell adhesion assay (40). In this assay, CdiA-expressing inhibitor cells are labeled with green fluorescent protein (GFP) and mixed at a 5:1 ratio with DsRed-labeled target bacteria. After the populations are allowed to interact, the cell suspension is analyzed by flow cytometry for events that exhibit both GFP and DsRed fluorescence (Fig. 3A), which are quantified as inhibitor-target cell aggregates. Control experiments show that <5% of target bacteria adhere nonspecifically to mock (CDI^−^) inhibitor cells (Fig. 3A and B). In contrast, cells that express CdiA bind to target bacteria in a receptor-dependent manner. CdiA^EC93^-expressing cells bound ~80% of *bamA*^Eco^-target bacteria but failed to aggregate with *bamA*^ECL^ targets (Fig. 3A and C). Similarly, inhibitor cells that express CdiA^EC536^ bind a substantial fraction of *ompC*^EC536^ target bacteria but exhibited only background adhesion with ∆*ompC* targets (Fig. 3A and D). As predicted, chimeric CdiA^EC93^ containing the putative receptor-binding region from CdiA^EC536^ recognized target cells in an *ompC*-dependent manner (Fig. 3A and E). Moreover, because this chimera was expressed at approximately the same level as CdiA^EC536^ (Fig. S2), the grafted domain appears to bind OmpF-OmpC with the same avidity as in its native context. These data, together with the *in vitro* binding results, indicate that the central regions of CdiA^EC93^ and CdiA^EC536^ are responsible for receptor recognition.

10.1128/mBio.00290-17.1 FIG S1  Alignment of representative class I, II, and III *E. coli* CdiA effectors. The predicted amino acid sequences of CdiA^EC93^ (AAZ57198.1), CdiA^EC536^ (WP_000554175.1), and CdiA^STECO31^ (WP_001385946.1) were aligned using Clustal Omega at http://www.uniprot.org. Domains and peptide motifs are outlined as determined by the EMBL-EBI InterPro protein sequence analysis site. Red boldface indicates the secretion signal sequence, green indicates the TPS transport domain, blue indicates FHA-1 peptide repeats (Pfam identifier PF05594), orange indicates FHA-2 peptide repeats (PF13332), yellow indicates the pretoxin-VENN domain (PF04829), and purple indicates the variable CdiA-CT toxins. Within the CdiA-CT region, black boldface indicates the toxin translocation domains. The receptor-binding regions are shown in black boldface. Download FIG S1, PDF file, 0.1 MB. Copyright © 2017 Ruhe et al. 2017 Ruhe et al. This content is distributed under the terms of the Creative Commons Attribution 4.0 International license.

10.1128/mBio.00290-17.2 FIG S2  CdiA immunoblot. Total urea-soluble protein from *E. coli* DL4259 cells expressing the indicated CdiA proteins was analyzed by immunoblotting using polyclonal antisera raised against the N-terminal TPS domain of CdiA^EC93^. Predicted molecular masses of CdiA effectors lacking N-terminal signal sequences are ~314 kDa for CdiA^EC93^, ~328 kDa for CdiA^EC536^, and ~320 kDa for CdiA^STECO31^. Download FIG S2, PDF file, 0.9 MB. Copyright © 2017 Ruhe et al. 2017 Ruhe et al. This content is distributed under the terms of the Creative Commons Attribution 4.0 International license.

### The CVR is required for OmpC-dependent toxin delivery.

**FIG 4:**
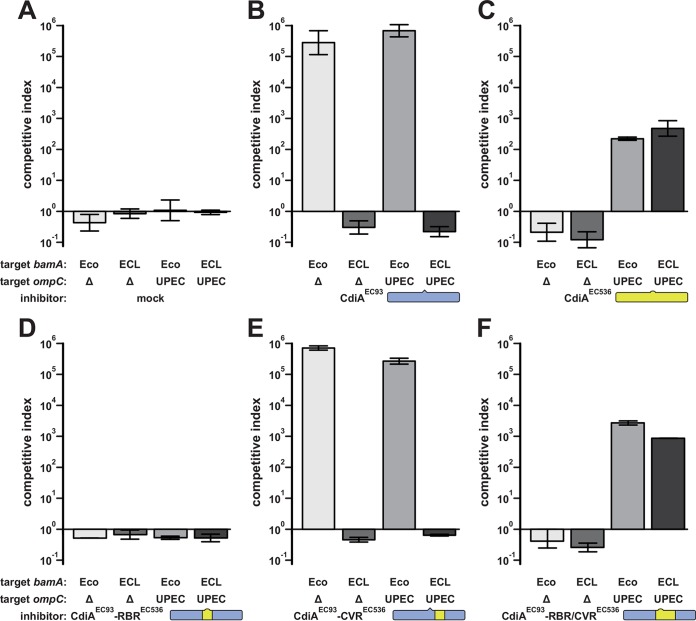
Competition cocultures. Target bacteria with the indicated *bamA* and *ompC* alleles were cocultured with inhibitor strains that express the following CdiA effectors: mock CDI^–^ (A), CdiA^EC93^ (B), CdiA^EC536^ (C), CdiA^EC93^ with the receptor-binding region (RBR) from CdiA^EC536^ (D), CdiA^EC93^ with the covarying region (CVR) from CdiA^EC536^ (E), and CdiA^EC93^ with both RBR and CVR from CdiA^EC536^ (F). Viable inhibitor and target cells were enumerated as CFU milliliter^−1^, and the competitive index was calculated as final inhibitor-to-target cell ratio divided by the initial ratio for each coculture. Competitive indices are reported as averages ± standard errors for two independent experiments. UPEC, uropathogenic *E. coli*.

Though the receptor-binding region chimera supported robust OmpC-dependent adhesion, this effector protein did not inhibit target cell growth in competition cocultures (Fig. 4D). In contrast, inhibitors expressing CdiA^EC93^ outcompeted target cells approximately 10^5^-fold in cocultures (Fig. 4B), and cells that deploy CdiA^EC536^ exhibited a greater than 100-fold advantage (Fig. 4C). In each instance, CDI^+^ inhibitors only outcompeted target cells that express the appropriate CdiA receptor (Fig. 4B and C). Together, these results suggest that the chimera is defective for toxin delivery. We noted that the CdiA^EC93^ region spanning Ala1910 to Gly2205 also diverges significantly with CdiA^EC536^ and that CdiA^EC536^ contains a 69-residue insertion in this region (Fig. 2 and Fig. S1). Because this region is conserved between CdiA^EC536^ homologues that bind OmpC-OmpF, we refer to this sequence as the “covarying region” (CVR). We explored the function of this sequence by replacing CdiA^EC93^ residues Ser1347 to Gly2205 with the receptor-binding and covarying regions of CdiA^EC536^ (Ala1345 to Gly2310) (Fig. 2). The resulting chimera was expressed stably (Fig. S2) and supported *ompC*-dependent cell-cell adhesion comparably to wild-type CdiA^EC536^ (Fig. 3A and G). Moreover, CdiA^EC93^ carrying the heterologous receptor-binding and covarying regions also inhibited target bacteria in an *ompC*-dependent manner (Fig. 4F). In contrast, another CdiA^EC93^ chimera containing only the covarying region from CdiA^EC536^ (Pro1669 to Gly2310) retained BamA-binding specificity (Fig. 3A and F) and inhibited target cells in a *bamA*^Eco^-dependent manner (Fig. 4E). Collectively, these data suggest that CdiA^EC536^ residues Pro1669 to Gly2310 may be important for toxin delivery through the OmpC-OmpF receptor pathway.

### Identification of the receptor for CdiA^STECO31^.

**FIG 5:**
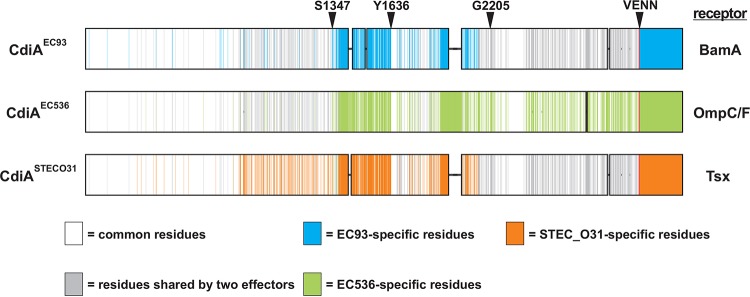
Comparison of class I, II, and III CdiA effectors. The CdiA^EC93^, CdiA^EC536^, and CdiA^STECO31^ proteins are presented schematically. Residues shared by all three proteins are shown in white, and residues shared by two CdiAs are shown in gray. Effector-specific residues are shown in blue (CdiA^EC93^), green (CdiA^EC536^), and orange (CdiA^STECO31^).

**FIG 6:**
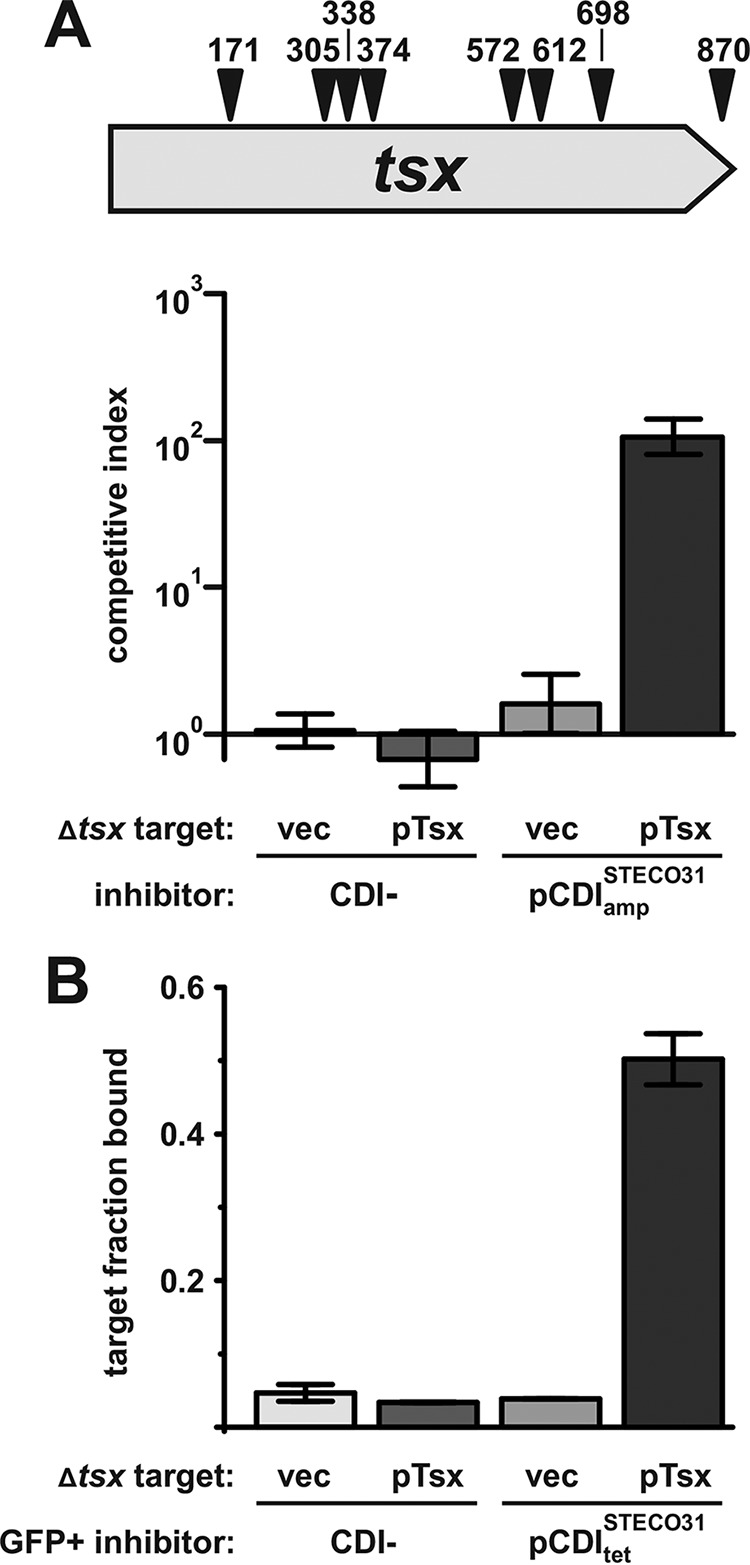
CdiA^STECO31^ uses Tsx as a receptor to bind target bacteria. (A) *E. coli* ∆*tsx* mutants are resistant to CdiA^STECO31^-mediated growth inhibition. Eight independent transposon insertions within *tsx* were identified in selections for CDI-resistant mutants. Inhibitor cells (CDI^STECO31^ or CDI^−^ mock) were cocultured with *tsx*^+^ or *Δtsx* target bacteria in shaking broth for 4 hours as described in Materials and Methods. Viable inhibitor and target cells were enumerated as CFU, and the competitive index was calculated as the final inhibitor-to-target cell ratio divided by the initial ratio. Competitive indices are presented as averages ± standard errors. (B) Tsx is required for CdiA^STECO31^-dependent cell-cell adhesion. The fraction of red fluorescent target bacteria bound to green fluorescent inhibitor cells was quantified for two independent experiments. Data are presented as averages ± standard errors.

We surveyed all identifiable *E. coli* CdiA effectors in the NCBI protein database and found that they segregate into at least four classes based on receptor-binding region sequences. CdiA^EC93^ defines the BamA-binding class I effectors, and CdiA^EC536^ is the paragon for class II effectors that recognize OmpC-OmpF. Class III CdiA proteins are closely related to class I and II over the two-partner secretion (TPS) transport domain and filamentous hemagglutinin (FHA)-peptide repeat regions but diverge significantly at the receptor-binding region (Fig. 5; see also Fig. S1). Class IV CdiA proteins appear to be more distantly related and share little sequence identity with the other three classes over the FHA-1 peptide repeat region (Fig. S3). To determine whether class III CdiA recognizes a unique receptor, we cloned the *cdiBAI* gene cluster from *E. coli* STEC_O31 and tested its activity in growth competitions. Class III CdiA^STECO31^ (NCBI reference sequence WP_001385946.1) carries a C-terminal EndoU RNase toxin domain, and cells deploying this effector outcompeted target bacteria after 4 hours of coculture (Fig. 6A). Having established growth inhibition activity, we used a genetic approach to identify the receptor for CdiA^STECO31^. We subjected target cells to *mariner* transposon mutagenesis and selected for CDI^STECO31^-resistant (CDI^r^) mutants. We isolated 12 CDI^r^ clones from three independently prepared mutant pools and identified transposon insertion sites. Four of the CDI^r^ mutants carried independent insertions in the *ptsG* gene, which encodes the fused IIB and IIC components of the glucose phosphotransferase system (PTS). We previously reported that *ptsG* null alleles also confer resistance to CDI toxins from *E. coli* 3006 and NC101 (14). CdiA-CT^STECO31^, CdiA-CT^3006^, and CdiA-CT^NC101^ all share the same “translocation” domain, which is thought to bind PtsG and mediate toxin transport from the periplasm into the target cell cytosol (14). The remaining eight CDI^r^ mutants were disrupted in *tsx* (Fig. 6A), which encodes an outer membrane nucleoside transporter. To confirm the role of Tsx in CDI^STECO31^, we tested target cells carrying a nonpolar ∆*tsx* mutation and found that they were resistant to growth inhibition (Fig. 6A). Moreover, complementation of ∆*tsx* target cells with plasmid-borne *tsx* restored sensitivity to growth inhibition (Fig. 6A). Finally, we used flow cytometry to demonstrate that Tsx is required for cell-cell adhesion between target bacteria and CdiA^STECO31^-expressing inhibitor cells (Fig. 6B). Together, these results show that the distinct receptor-binding region of class III CdiA^STECO31^ recognizes Tsx.

10.1128/mBio.00290-17.3 FIG S3  Alignment of representative class I and IV *E. coli* CdiA effectors. The predicted amino acid sequences of CdiA^EC93^ (AAZ57198.1) and CdiA_2_^STECO31^ (WP_001081258.1) were aligned using Clustal Omega at http://www.uniprot.org. Domains and peptide motifs are outlined as determined by the EMBL-EBI InterPro protein sequence analysis site. Red boldface indicates the secretion signal sequence, green indicates the TPS transport domain, blue indicates FHA-1 peptide repeats (Pfam identifier PF05594), orange indicates FHA-2 peptide repeats (PF13332), yellow indicates the pretoxin-VENN domain (PF04829), and purple indicates the variable CdiA-CT toxins. The receptor-binding region of CdiA^EC93^ is shown in black boldface. Download FIG S3, PDF file, 0.1 MB. Copyright © 2017 Ruhe et al. 2017 Ruhe et al. This content is distributed under the terms of the Creative Commons Attribution 4.0 International license.

## DISCUSSION

The results presented here show that CdiA effectors from *E. coli* strains bind target bacteria using at least three cell surface receptors. Receptor specificity for the different CdiA classes is determined by an unannotated stretch of ~300 residues located between the FHA-1 and FHA-2 peptide repeat regions (see Tables S1, S2, S3, and S4 in the supplemental material). CdiA^EC93^ is the paragon for class I effectors, which bind specifically to BamA from *E. coli* (28). Class II effectors recognize heterotrimeric OmpC-OmpF osmoporins (31), and here, we show that class III effectors bind the outer membrane nucleoside transporter Tsx. The receptor-binding regions of class I, II, and III effectors share 24 to 27% pairwise sequence identity, and each domain contains a conserved central FHA-1 element (Fig. S1). These observations suggest that the classes diverged from a common ancestral sequence and that the three receptor-binding domains adopt similar conformations. The domains also exhibit some mutational drift within each class, though it is not clear whether these minor sequence variations influence receptor specificity. Class I effectors carry nine different receptor-binding sequences that arise from combinations of 17 missense changes (Table S1). Class II receptor-binding regions are less polymorphic with only nine missense variations (Table S2), and there are 14 distinct class III receptor-binding sequences (Table S3). Class IV *E. coli* CdiA effectors have diverged from the other three classes but still share the same overall domain structure (Fig. S3). Given the similarities in effector architecture, we predict that the receptor-binding domain of class IV effectors also resides between the FHA-1 and FHA-2 regions. Notably, class IV *cdi* gene clusters also encode a predicted HlyC-type lysyl acyltransferase (14). The functional significance of this acyltransferase has not yet been explored, but it presumably modifies CdiA and/or CdiB to promote cell adhesion and growth inhibition activities. Additionally, the database contains three CdiA proteins from *E. coli* isolates SWW33 (WP_001764992.1), upec-172 (WP_052432358.1), and 696_ECOL (WP_049080366.1) that do not fit into the four major classes. These proteins are most closely related to class IV CdiA but have longer FHA-1 repeat regions and diverge over the central (putative) receptor-binding region (Fig. S4). Moreover, the three effectors are encoded by *cdi* gene clusters that lack the characteristic *hlyC* acyltransferase of class IV loci. These observations suggest that these three CdiA proteins constitute a fifth effector class that probably recognizes yet another cell surface receptor.

10.1128/mBio.00290-17.4 FIG S4  Alignment of representative class IV and V *E. coli* CdiA effectors. The predicted amino acid sequences of CdiA_2_^STECO31^ (WP_001081258.1) and CdiA^SWW33^ (WP_001764992.1) were aligned using Clustal Omega at http://www.uniprot.org. Domains and peptide motifs for CdiA_2_^STECO31^ are outlined as determined by EMBL-EBI InterPro, but there are currently no available annotations for CdiA^SWW33^. Red boldface indicates the secretion signal sequence, green indicates the TPS transport domain, blue indicates FHA-1 peptide repeats (Pfam identifier PF05594), orange indicates FHA-2 peptide repeats (PF13332), yellow indicates the pretoxin-VENN domain (PF04829), and purple indicates the variable CdiA-CT toxins. Within the CdiA-CT region, black boldface indicates the toxin translocation domain. Download FIG S4, PDF file, 0.05 MB. Copyright © 2017 Ruhe et al. 2017 Ruhe et al. This content is distributed under the terms of the Creative Commons Attribution 4.0 International license.

10.1128/mBio.00290-17.5 TABLE S1  Predicted class I CdiA proteins encoded by *E. coli* isolates. The nonredundant protein database was searched for full-length CdiA effectors based on homology to the receptor-binding region of CdiA^EC93^ (AAZ57198.1). Missense changes with respect to CdiA^EC93^ are indicated by orange-filled boxes. Download TABLE S1, PDF file, 0.04 MB. Copyright © 2017 Ruhe et al. 2017 Ruhe et al. This content is distributed under the terms of the Creative Commons Attribution 4.0 International license.

10.1128/mBio.00290-17.6 TABLE S2  Predicted class II CdiA proteins encoded by *E. coli* isolates. The nonredundant protein database was searched for full-length CdiA effectors based on homology to the receptor-binding region of CdiA^EC536^ (WP_000554175.1). Missense changes with respect to CdiA^EC536^ are indicated by orange-filled boxes. Download TABLE S2, PDF file, 0.05 MB. Copyright © 2017 Ruhe et al. 2017 Ruhe et al. This content is distributed under the terms of the Creative Commons Attribution 4.0 International license.

10.1128/mBio.00290-17.7 TABLE S3  Predicted class III CdiA proteins encoded by *E. coli* isolates. The nonredundant protein database was searched for full-length CdiA effectors based on homology to the receptor-binding region of CdiA_1_^STECO31^ (WP_001385946.1). Missense changes with respect to CdiA_1_^STECO31^ are indicated by orange-filled boxes. Download TABLE S3, PDF file, 0.02 MB. Copyright © 2017 Ruhe et al. 2017 Ruhe et al. This content is distributed under the terms of the Creative Commons Attribution 4.0 International license.

10.1128/mBio.00290-17.8 TABLE S4  Predicted class IV CdiA proteins encoded by *E. coli* isolates. The nonredundant protein database was searched for full-length CdiA effectors based on homology to CdiA_2_^STECO31^ (WP_001081258.1). Download TABLE S4, PDF file, 0.04 MB. Copyright © 2017 Ruhe et al. 2017 Ruhe et al. This content is distributed under the terms of the Creative Commons Attribution 4.0 International license.

Sequence alignments of class I, II, and III CdiA effectors predict that their receptor-binding domains are interchangeable. This hypothesis is supported by data showing that chimeric CdiA^EC93^ carrying a class II receptor-binding region is endowed with OmpC-binding activity. Though this chimera appears to bind OmpC-OmpF with the same avidity as wild-type CdiA^EC536^, it does not inhibit target bacteria, indicating that toxin delivery function is compromised. Growth inhibition activity is restored when the covarying region is grafted together with the receptor-binding region. The latter result suggests that the covarying region of CdiA^EC536^ is critical for toxin delivery through the OmpC pathway. However, chimeric CdiA^EC93^ carrying the covarying region from CdiA^EC536^ inhibits target bacteria in a BamA^Eco^-dependent manner, showing that this sequence is not solely dedicated to the OmpC-OmpF pathway. In the context of domain modularity, these observations raise the possibility that specific toxin families may be excluded from certain receptor-mediated delivery pathways. Surveys of CdiA proteins show that some toxin families tend to be paired with specific receptor-binding domains. For example, Ntox28 RNase domains are found only on class II effectors in *E. coli* (Table S2). However, Ntox28 toxins are very effective at killing target bacteria when experimentally grafted onto class I CdiA^EC93^ (13, 14, 41). Thus, the apparent bias in naturally occurring effectors does not necessarily reflect domain incompatibility. In fact, we have shown that CdiA^EC93^ can deploy over a dozen heterologous toxin domains (9, 13, 14, 19–21, 41, 42), even though many of the grafted CdiA-CT sequences are not found on class I effectors. Moreover, the database shows significant combinatorial flexibility for some toxin families. For example, endonuclease NS_2 toxin domains are associated with each of the four major effector classes in *E. coli* (Tables S1 to S4). Comprehensive analysis of *E. coli* CdiA proteins reveals that each effector class is associated with multiple different toxin families (Tables S1 to S4). Taken together, these observations suggest that new CdiA effectors are assembled through genetic recombination to exchange receptor-binding domains and toxic payloads.

**FIG 7:**
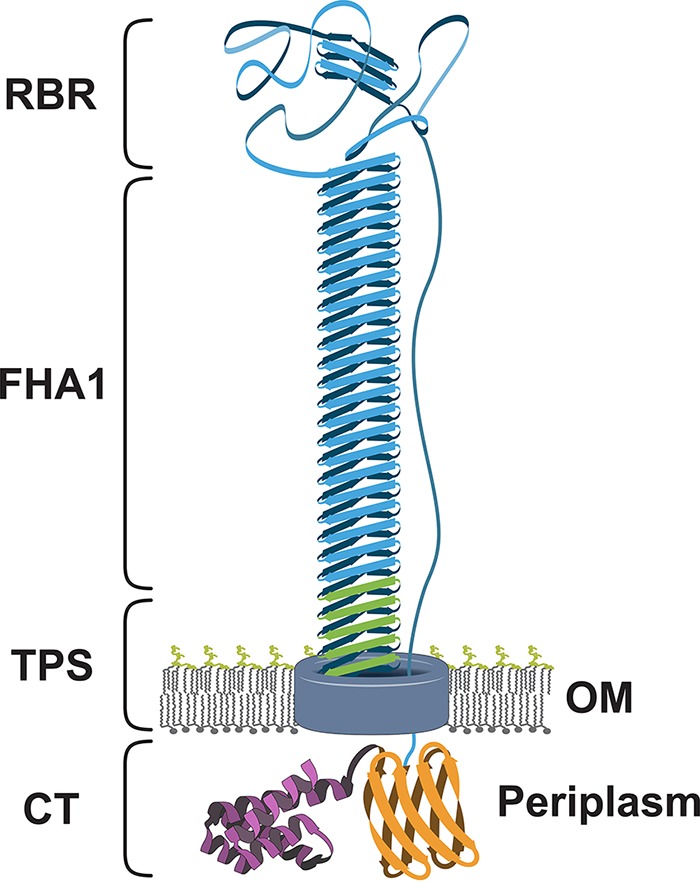
Topological model for CdiA. The constituent domains of cell surface CdiA are labeled and color coded as in Fig. 1. CdiB is represented as a barrel in the outer membrane (OM). The proposed model is based on unpublished protease protection data and the work of Noël et al. (48).

The location of the receptor-binding region has implications for the structure and presentation of CdiA on the inhibitor cell surface. CdiA effectors are thought to be structurally similar to the FhaB adhesins of *Bordetella* species because the protein families share related domain architectures (3, 12). FhaB is synthesized initially as a 370-kDa precursor, from which the C-terminal “prodomain” is removed to yield a mature filamentous hemagglutinin (FHA) fragment of ~220 kDa (43, 44). FHA is monomeric and extends about 50 nm in length (45). The central shaft of FHA corresponds to the FHA-1 peptide repeat region, which is predicted to form a right-handed β-helix with a 4.8-Å pitch per 20-residue repeat (45, 46). According to this model, the FHA-1 repeat region of CdiA^EC93^ should form a filament 30 to 35 nm in length. It is less clear that the CdiA FHA-2 repeat region is homologous to the FhaB prodomain. Though the prodomain contains a short FHA-2 region (Phe2927 to Gly3086), CdiA and FhaB share little sequence identity over this region. Moreover, FhaB lacks a C-terminal toxin domain and does not mediate interbacterial competition (47). Nevertheless, FhaB and CdiA are processed in a similar manner, with immunoblot analysis revealing a stable N-terminal CdiA fragment of ~230 kDa (see Fig. S2). This processed fragment almost certainly retains adhesin activity, because truncated CdiA^EC93^ lacking the FHA-2 and CdiA-CT regions is still exported to the cell surface and mediates BamA^Eco^-dependent adhesion (38). These observations raise questions about the location of the FHA-2/CdiA-CT regions relative to the cell surface. Current models assume that the CdiA C terminus projects away from the inhibitor cell to facilitate toxin transfer to target bacteria. However, we propose that the receptor-binding domain should be positioned at the distal tip of the filament (Fig. 7), where it can easily interact with target bacteria. Studies on FhaB biogenesis from the Cotter lab provide further support for the topological model presented in Fig. 7 (48). Their work indicates that the C-terminal prodomain is retained in the periplasm during FhaB export and processing. Thus, both N and C termini of FhaB remain within an intracellular compartment while the FHA-1 region assembles into a β-helix on the cell surface (48). It is not clear how the FhaB chain is exported as a tethered loop; however, their work predicts that the CdiA receptor-binding domain should be located at the distal tip of the FHA-1 repeat filament. Further, the model suggests that the FHA-2/CdiA-CT region is sequestered within the inhibitor cell periplasm prior to receptor recognition (Fig. 7). The latter prediction is consistent with unpublished data from our laboratories showing that the C-terminal region is protected from extracellular protease, whereas the N-terminal TPS domain and FHA-1 region are rapidly degraded by this treatment. If this model is correct, then receptor recognition must induce an extraordinary change in CdiA conformation to transfer the CdiA-CT toxin domain from the inhibitor cell periplasm into the target bacterium.

## MATERIALS AND METHODS

### Bacteria, growth conditions, and competition cocultures.

Bacteria used in this study are listed in Table 1. All strains were grown at 37°C in lysogeny broth (LB) or on LB agar unless otherwise indicated. Media were supplemented with antibiotics at the indicated concentrations: ampicillin (Amp), 150 µg ml^−1^; chloramphenicol (Cm), 33 µg ml^−1^; kanamycin (Kan), 50 μg ml^−1^; rifampin (Rif), 200 µg ml^−1^; and tetracycline (Tet), 15 μg ml^−1^. The ∆*ompC*::*kan* allele from the Keio collection (49) was amplified using oligonucleotides ompC(UPECswap)-Sac (5′-TTT GAG CTC CTG GAA ATT ATG CGG ATG) and ompC(UPECswap)-Kpn (5′-TTT GGT ACC CCA CAG ATT CAC CAG C), and the product was recombined into *E. coli* CH9597 cells carrying plasmid pSIM5 (50). The *ompC*^EC536^-*kan* allele (31) was introduced into *E. coli* CH9597 in the same manner. Kan resistance cassettes were subsequently removed with FLP recombinase. Plasmid exchange was used to displace pZS21*amp*-*bamA*^+^ with Kan-resistant pZS21-*bamA* or pZS21-*bamA*^ECL^ (28). The ∆*tsx*::*kan* gene disruption was transduced into *E. coli* EPI100 that expresses *recA* from pCH9674, which was subsequently cured by growing the resulting strain at 42°C. For competition cocultures, inhibitor and target bacteria were adjusted to an optical density at 600 nm (OD_600_) of 0.05 and then mixed at a 10:1 ratio and incubated in shaking LB medium for 4 h at 37°C. Cell suspensions were diluted into 1× M9 salts and plated onto antibiotic selective LB-agar to enumerate inhibitor and target cells as CFU per milliliter. Competitive indices were calculated as the ratio of target to inhibitor cells at 4 h divided by the ratio at the beginning of coculture.

**TABLE 1:** Bacterial strains

Strain	Description^a^	Source or reference
EPI100	F^−^ *mcrA* Δ(*mrr-hsdRMS-mcrBC*) φ80d*lacZ∆*M15 *∆lacXcZ*∆M15 *∆lacX recA1 endA1 araD139 Δ*(*ara*, *leu*)*7697 galU galK* λ^−^ *rpsL nupG* Str^r^	Epicentre
MFD*pir*	MG1655 RP4-2-Tc::[∆Mu1::*aac(3)IV*-∆*aphA*-∆*nic*35-∆Mu2::*zeo*] *dapA*::(*erm*-*pir*) ∆*recA* Apr^r^ Zeo^r^ Erm^r^	55
DY378	W3110 λ*cI857 Δ*(*cro-bioA*)	50
DL4259	*E. coli* MC4100 that expresses GFP-*mut3*	16
CH2016	X90 (DE3) *∆rna ∆slyD*::*kan* Rif^r^ Kan^r^	56
CH7175	EPI100 ∆*wzb* Str^r^	19
CH8119	DH5α *pir*^+^	Biomedal s.l. (Spain)
CH9597	EPI100 *∆bamA*::*cat* pZS21*amp*-*bamA*^+^ Str^r^ Cm^r^ Amp^r^	28
ZR343	EPI100 *∆bamA*::*cat* ∆*ompC* pZS21-*bamA*^+^ Str^r^ Cm^r^ Kan^r^	This study
ZR344	EPI100 *∆bamA*::*cat ∆ompC* pZS21-*bamA*^ECL^ Str^r^ Cm^r^ Kan^r^	This study
ZR345	EPI100 *∆bamA*::*cat ompC*^EC536^ pZS21-*bamA*^+^ Str^r^ Cm^r^ Kan^r^	This study
ZR346	EPI100 *∆bamA*::*cat ompC*^UPEC536^ pZS21-*bamA*^ECL^ Str^r^ Cm^r^ Kan^r^	This study
ZR372	EPI100 *∆wzb* Δ*tsx*::*kan* Str^r^ Kan^r^	This study
ZR376	EPI100 *∆wzb Δtsx* Str^r^	This study
ZR412	EPI100 *∆bamA*::*cat* ∆*ompC*::*kan* pZS21*amp*-*bamA*^+^ Str^r^ Cm^r^ Kan^r^ Amp^r^	This study
ZR413	EPI100 *∆bamA*::*cat ∆ompC*::*kan* pZS21*amp*-*bamA*^ECL^ Str^r^ Cm^r^ Kan^r^ Amp^r^	This study

a Abbreviations: Amp^r^, ampicillin resistant; Apr^r^, apramycin resistant; Cm^r^, chloramphenicol resistant; Erm^r^, erythromycin resistant; Kan^r^, kanamycin resistant; Rif^r^, rifampin resistant; Str^r^, streptomycin resistant; Zeo^r^, zeocin resistant.

### Plasmid constructions.

Plasmids used in this study are listed in Table 2. The *bamA*^Eco^ gene from *E. coli* MG1655 was amplified with oligonucleotides bamA(∆ss)-Spe (5′-TTT ACT AGT GAA GGG TTC GTA GTG AAA G) and bamA-rev-Sal (5′-TCC TTT GTC GAC AAC ACT TAC CAG GTT TTA CC), digested with SpeI/SalI, and ligated to SpeI/XhoI-digested plasmid pCH7277. The resulting pCH9216 construct overproduces BamA^Eco^ under the control of the bacteriophage T7 promoter. The *bamA*^ECL^ gene from *Enterobacter cloacae* ATCC 13047 was amplified with primer pair bamA(∆ss)-Spe and Ecloa-bamA-Sal-rev (5′-TTT GTC GAC GAG AAT TAC CAG GTT TTA CC) and cloned in the same manner. Fragments of *cdiA*^EC93^ were amplified from plasmid pDAL660∆1-39 using the following primer pairs: EC93-cdiA(R1358)-Spe (5′-TTT ACT AGT CGT TCC GGT AAT ATT GAA ACC) and EC93-cdiA(R2123)-Xho (5′-TTT CTC GAG TTA CCG GGT GGT CTG ATG GC), EC93-cdiA(R1358)-Spe and EC93-cdiA(F1646)-Xho (5′-TTT CTC GAG CTA AAA ATA ACC ATT GTT ACC GGA TGG), EC93-cdiA(G1953)-Spe (5′-TTT GAG CTC ACT AGT GGT GAC AGC GTG TTA CTG G) and EC93-cdiA(N2556)-Xho (5′-TTT CTC GAG GAT CCT AAT TGC TTT TAA TTT TGT CCT GGC), EC93-cdiA(N2556)-Spe (5′-TTT GAG CTC ACT AGT AAT TAC GAT TCT GTC CGG GAA C) and cdiA(V2727)-Xho (5′-TTT CTC GAG GAT CCT AGA CGA TGT TAC TCA TCT GAC C), and EC93-cdiA(Q2681)-Spe (5′-TTA ACT AGT CAG GAT ATT GCC GGG CTC) and EC93-cdiA(A2909)-Xho (5′-TTA CTC GAG TTA TGC ATT ATT CTC AAC CGA G). The resulting PCR products were digested with SpeI/XhoI and ligated to plasmid pCH7277 to generate constructs that overproduce His_6_-tagged CdiA^EC93^ fragments.

**TABLE 2:** Plasmids

Plasmid	Description^a^	Source or reference
pWEB-TNC	Cosmid cloning vector; Amp^r^ Cm^r^	Epicentre
pCP20	Heat-inducible expression of FLP recombinase; Cm^r^ Amp^r^	57
pSC189	Mobilizable plasmid with R6Kγ replication origin; carries the *mariner* transposon containing kanamycin resistance cassette; Amp^r^ Kan^r^	58
pRE118	Vector plasmid for allelic exchange; Kan^r^	52
pSIM5	Heat-inducible expression of the phage λ Red recombinase proteins; Cm^r^	59
pSIM6	Heat-inducible expression of the phage λ Red recombinase proteins; Amp^r^	59
pDsRedExpress2	Constitutive expression of DsRed; Amp^r^	Clontech
pZS21-*bamA*^+^	pZS21 derivative that expresses *bamA*^Eco^; Kan^r^	60
pZS21-*bamA*^ECL^	Expresses *bamA*^ECL^ from *Enterobacter cloacae* ATCC 13047; Kan^r^	28
pZS21*amp*-*bamA*^+^	pZS21*amp* derivative that expresses *E. coli bamA*; Amp^r^	24
pZS21*amp*-*bamA*^ECL^	pZS21*amp* derivative that expresses *E. cloacae bamA*; Amp^r^	28
pDAL660∆1-39	Constitutively expresses the *cdiBAI*^EC93^ gene cluster; Amp^r^ Cm^r^	3
pDAL7718	pDAL660∆1-39 derivative that expresses chimeric CdiA^EC93^ with residues Ser1347 to Tyr1636 replaced with Ala1345 to Trp1668 from CdiA^EC536^; Amp^r^ Cm^r^	This study
pDAL7720	pDAL660∆1-39 derivative that expresses chimeric CdiA^EC93^ with residues Ser1347 to Gly2205 replaced with residues Ala1345 to Gly2310 from CdiA^EC536^; Amp^r^ Cm^r^	This study
pDAL7912	pDAL660∆1-39::*kan-sacB*; Amp^r^ Cm^r^ Kan^r^	This study
pDAL7936	pDAL660∆1-39 derivative that expresses chimeric CdiA^EC93^ with residues Pro1637 to Gly2205 replaced with residues Pro1669 to Gly2310 from CdiA^EC536^; Amp^r^ Cm^r^	This study
pCH450	pACYC184 derivative with *E. coli araBAD* promoter for arabinose-inducible expression; Tet^r^	51
pCH7277	pSH21::*arfA*, contains a SpeI site to generate in-frame N-terminal His_6_ fusion proteins; Amp^r^	61
pCH8619	pSH21::*cdiA*(Q2681–A2909); Amp^r^	This study
pCH9216	pSH21::(*∆ss*)*bamA*^Eco^; overproduces *E. coli* BamA lacking the signal sequence peptide; Amp^r^	This study
pCH9231	pSH21::(*∆ss*)*bamA*^ECL^; overproduces *E. cloacae* BamA lacking the signal sequence peptide; Amp^r^	This study
pCH9674	pSIM6 derivative in which the phage λ *red* genes have been replaced with *E. coli recA*; Amp^r^	This study
pCH9718	pSH21::*cdiA*(R1358–R1646); Amp^r^	This study
pCH10235	pSH21::*cdiA*(R1358–R2123); Amp^r^	This study
pCH10316	pSH21::*cdiA*(N2556–V2727); Amp^r^	This study
pCH10319	pSH21::*cdiA*(G1953–N2556); Amp^r^	This study
pCH12352	pCH450::*cdiBAI*^EC536^; Tet^r^	This study
pCH13602	pCH450::*cdiBAI*^STECO31^; Tet^r^	This study
pCH13603	pCH450::*tsx*; Tet^r^	This study
pCH13604	pET21b::*cdiBAI*^STECO31^; Amp^r^	This study

a Abbreviations: Amp^r^, ampicillin resistant; Spc^r^, spectinomycin resistant; Kan^r^, kanamycin resistant; Tmp^r^, trimethoprim resistant; Tet^r^, tetracycline resistant.

The *cdiBAI*^EC536^ gene cluster (ordered loci, ECP_4581, ECP_4580, and ECP_4579) was amplified from *E. coli* 536 genomic DNA using oligonucleotides UPEC-cdiB-Not-for (5′-TTT GCG GCC GCC ATT TAT AAG AAT ACG CCG CTT CG) and UPEC-cdiI-XhoI-rev (5′-TTT CTC GAG CCA CCA TCA GGC TGG AC). The product was digested with NotI/XhoI and ligated to plasmid pCH450 (51). The *cdiBAI*^STECO31^ gene cluster (ordered loci, BE44_RS05220, BE44_RS05225, and BE44_RS0125610) was amplified from *E. coli* STEC_O31 genomic DNA using oligonucleotides J2M139-CDI-Not-for (5′-TTT GCG GCC GCA ATG TCT GGT TGT GGC AGG) and J2M139-CDI-Xho-rev (5′-TTT CTC GAG TGG CCG GAA TCT TTA CTC AG) (http://www.uniprot.org/uniparc/UPI00026E100C). The product was digested with NotI/XhoI and ligated to plasmids pET21b and pCH450. The *E. coli tsx* gene was amplified with tsx-Not-for (5′-TTT GCG GCC GCG AAT TCG GGA TTT TCA AAC AGT GGC ATA C) and tsx-Xho-rev (5′-TTT CTC GAG TCT AGA AAA TCC CGG CAT TTT CAT AAT CAG) and ligated to plasmid pCH450 using NotI and XhoI restriction sites. The *E. coli recA* gene was amplified with recA-BglII (5′-TGC AGA TCT TGT GGC AAC AAT TTC TAC) and recA-Xma (5′-GCG ACC CGG GTG TAT CAA ACA AGA CG) and ligated to BglII/XmaI-digested plasmid pSIM6 to generate pCH9674.

Plasmid pDAL7912 was constructed by introducing Kan resistance and counterselectable *sacB* genes into pDAL660∆1-39. The *sacB* and *kan* genes were amplified from plasmid pRE118 (52) with primers EC93-1953-sacB-for (5′-CGC CGT TTC TGC CGG T CCC GTA GTC TGC AAA TCC) and EC93-1972-sacB-rev (5′-GCC ATA GCG ACG ACG TTC TCC GAA AAT GCC AAT AGG ATA TCG GC). Two *cdiA*^EC93^ fragments were then amplified with primer pairs EC93-Chimera-for (5′-ACG TTA AAG GAA CCA CGC TG)/EC93-1953-sacB-rev (5′-GGA TTT GCA GAC TAC GGG ACC GGC AGA AAC GGC G) and EC93-1972-sacB-for (5′-GCC GAT ATC CTA TTG GCA TTT TCG GAG AAC GTC GTC GCT ATG GC)/EC93-Chimera-rev (5′-GAA AGT CAC AGC AGA TGT CGG). The three fragments were combined by overlap-extension PCR (OE-PCR) using primer pair EC93-chimera-for/EC93-chimera-rev. The resulting product was introduced into plasmid pDAL660∆1-39 by Red-mediated recombination, and the recombinant pDAL660∆1-39::*sacB-kan* plasmid was selected on Kan-supplemented LB agar.

Chimeric *cdiA*^EC93^ genes were constructed by Red-mediated recombination and sucrose counterselection. The receptor-binding region of *cdiA*^EC536^ (encoding residues Ala1345 to Trp1668) was amplified with UPEC1345A (5′-GTC TGT GGG TAC AGA AGG ACG CTT CCG GCG GTG CAA ACA) and UPEC1668W (5′-TGT TAC CGG ATG GCA GGG GCC AGT CAT CAC TGA TAC CGG). The covarying region of *cdiA*^EC536^ (encoding residues Pro1669 to Gly2310) was amplified with UPEC1669P (5′-GGG CAA GTG TAA GCA GCT ATC CAC TGC CTT CCG GCA ACA A) and UPEC2310G (5′-TCG TGC GTT GTC TTA CTG CTG CCA ATG GTG AAA CCA ATA CC). The combined receptor-binding and covarying regions were amplified with primer pair UPEC1345A/UPEC2310G. Homology regions from *cdiA*^EC93^ were amplified with EC93-Chimera-for/UPEC1345-rev (5′-TGT TTG CAC CGC CGG AAG CGT CCT TCT GTA CCC ACA GAC) encoding Asp1124 to Asp1346, EC93-Chimera-for/UPEC1669-rev (5′-TTG TTG CCG GAA GGC AGT GGA TAG CTG CTT ACA CTT GCC C) encoding Asp1124 to Tyr1636, UPEC1668-for (5′-CCG GTA TCA GTG ATG ACT GGC CCC TGC CAT CCG GTA ACA)/EC93-Chimera-rev encoding Pro1637 to Gly2508, and UPEC2311-for (5′-GGT ATT GGT TTC ACC ATT GGC AGC AGT AAG ACA ACG CAC GA)/EC93-Chimera-rev encoding Ser2207 to Gly2508. The resulting fragments were joined to *cdiA*^EC536^ amplicons using OE-PCR with primer pair EC93-Chimera-for/EC93-Chimera-rev. The final products were introduced into plasmid pDAL7912 by Red-mediated recombination, and chimeric recombinants were selected on no-salt LB agar supplemented with Amp and 5% sucrose. The identities of all plasmid constructs were confirmed by DNA sequence analysis.

### Protein purification.

BamA^Eco^ and BamA^ECL^ were overproduced in *E. coli* CH2016 grown at 37°C in LB medium supplemented with Amp and 1.5 mM IPTG (isopropyl-β-d-thiogalactopyranoside). Though the plasmid constructs encode N-terminal His_6_ epitopes, we found that these tags were removed, precluding the use of Ni^2+^ affinity chromatography. Therefore, we isolated BamA proteins from insoluble inclusion bodies. Bacteria were harvested by centrifugation, and the cell pellets were suspended in 5 ml of BugBuster reagent and incubated on a rotisserie at room temperature for 20 min. Next, cells were harvested by centrifugation at 6,000 × *g* for 10 min, and the supernatant was decanted to remove soluble proteins. Cells were then resuspended in 5 ml of BugBuster reagent and broken by three passages through a French press. Lysates were diluted with 25 ml deionized water and vortexed vigorously. Inclusion bodies were collected by centrifugation at 15,000 × *g* for 20 min and washed three times with 5 ml of 0.1× BugBuster solution. Isolated inclusion bodies were dissolved in 0.5 ml of urea lysis buffer (8 M urea, 10 mM Tris-HCl [pH 8.0], 150 mM NaCl, 20 mM imidazole, 0.05% Triton X-100). Residual His_6_-tagged BamA was removed by overnight incubation with urea lysis buffer-equilibrated Ni^2+^-nitrilotriacetic acid (NTA) agarose resin. Unbound protein was collected by centrifugation through an 0.45-μm nylon Costar Spin-X column and diluted into 10 mM Tris-HCl (pH 8.0)-0.5% Triton X-100. Refolding reaction mixtures were incubated on a rotisserie for 3 days at ambient temperature, followed by 3 weeks at 4°C. More than 95% of BamA was refolded as determined by heat-modifiable gel mobility as previously described (53).

His_6_-tagged CdiA^EC93^ fragments were overproduced in *E. coli* CH2016 grown at 37°C in LB medium supplemented with Amp and 1.5 mM IPTG. Bacteria were harvested by centrifugation, and the cell pellets were frozen at −80°C and then resuspended directly in 15 ml urea lysis buffer. Cells were disrupted by two freeze-thaw cycles. Insoluble debris was removed through two rounds of centrifugation at 15,000 × *g* for 20 min. Ni^2+^-NTA agarose resin was equilibrated in urea lysis buffer and added to each clarified lysate, and the mixtures were incubated on a rotisserie for 90 min at ambient temperature. The resin was collected by centrifugation at 3,000 × *g* for 30 s, resuspended in 5 ml urea lysis buffer, and transferred to a fresh tube. After three washes with 5 ml urea lysis buffer, resin-bound His_6_-CdiA^EC93^ fragments were refolded with three washes of 5 ml native lysis buffer (10 mM Tris-HCl [pH 8.0], 150 mM NaCl, 30 mM imidazole, 0.05% Triton X-100). After the final wash, resins were resuspended in 250 μl of binding buffer (10 mM Tris-HCl [pH 8.0], 150 mM NaCl, 30 mM imidazole, 0.5% Triton X-100) for use in BamA-binding experiments.

### BamA-binding assays.

Refolded BamA^Eco^ and BamA^ECL^ were diluted to 10 μM in 1 ml of binding buffer. Ni^2+^-NTA agarose resin (20 µl) containing bound His_6_-CdiA^EC93^ fragments was added to the BamA solutions, and the mixtures were incubated on a rotisserie for 90 min at 4°C. Resins were collected by centrifugation at 3,000 × *g* for 30 s, resuspended in 1 ml of binding buffer, and transferred to a fresh tube. The resins were washed three times with binding buffer and then eluted with binding buffer supplemented with 25 mM EDTA. Eluted proteins were run on SDS-10% polyacrylamide gels and blotted onto nitrocellulose for immunoblot analysis using polyclonal antibodies raised against BamA^Eco^. Blots were visualized using IRDye 680 (Li-Cor)-labeled anti-rabbit secondary antibodies and an Odyssey infrared imager as described previously (54).

### Cell-cell adhesion assays.

BamA^Eco^-binding studies were conducted using *E. coli* strain DL4259, which expresses *gfp-mut3* from the *papBA* promoter (19). *E. coli* DL4259 cells were transformed with pDAL660∆1-39 (CdiA^EC93^), pCH12352 (CdiA^EC536^), pDAL7718 (CdiA^EC93^-RBR^EC536^), pDAL7936 (CdiA^EC93^-CVR^EC536^), pDAL7720 (CdiA^EC93^-RBR/CVR^EC536^), or pCH13604 (CDI^STECO31^), and the resulting strains were grown at 37°C in LB medium (supplemented with Amp or Tet) until the cells developed fluorescence. The expression of each CdiA effector was assessed by immunoblot analysis of total urea-soluble protein using polyclonal antibodies raised against the N-terminal TPS transport domain of CdiA^EC93^ (38). Target cells were fluorescently labeled with DsRed using plasmid pDsRedExpress2. For Tsx-dependent adhesion experiments, the target cells were grown overnight in LB supplemented with Tet, Amp, 0.4% arabinose, and 1 mM IPTG to induce expression of Tsx and DsRed prior to mixing with inhibitors. GFP-labeled inhibitor cells were mixed at a 5:1 ratio with DsRed-labeled target bacteria at a final OD_600_ of 0.2. Cell suspensions were shaken at 30°C for 20 min, diluted into 1× PBS, vortexed briefly, and then analyzed on an Accuri C6 flow cytometer using FL1 (533/30 nm, GFP) and FL2 (585/40 nm, DsRed) fluorophore filters. The fraction of target bacteria bound to inhibitor cells was calculated as the number of dual green/red fluorescent events divided by the total number of red fluorescent events.

### Transposon library construction and selection for CDI^r^ mutants.

The *mariner* transposon was introduced into *E. coli* CH7175 cells through conjugation with *E. coli* MFD*pir* donor cells carrying plasmid pSC189. Donor cells were grown to mid-log phase in LB medium supplemented with Amp and 30 µM diaminopimelic acid. Donors and recipients were mixed and incubated on LB agar for 4 h at 37°C. Cell mixtures were harvested with a sterile swab, diluted into 1× M9 medium, and plated onto Kan-supplemented LB agar to select transposon mutants. The following day, the mutant pool (~100,000 Kan^r^ colonies) was harvested into 1 ml of 1× M9 medium. Mutant pools were cocultured with *E. coli* EPI100 cells carrying plasmid pCH13604 in LB medium overnight at 37°C, and target bacteria were selected on Kan-supplemented LB agar. The surviving target cell colonies were harvested into 1× M9 medium and subjected to two additional cycles of coculture selection. Four CDI^r^ clones were randomly selected from each of the three independently prepared transposon mutant pools. Transposon insertion sites were identified by rescue cloning. Chromosomal DNA from each CDI^r^ mutant was digested with NspI overnight at 37°C. The restriction endonuclease was inactivated at 65°C for 20 min, and reaction mixtures were supplemented with ATP and T4 DNA ligase for overnight incubation at 16°C. The ligated DNA was electroporated into *E. coli* DH5α *pir*^+^ cells. Plasmids were isolated from resulting Kan-resistant transformants, and transposon insertion junctions were identified by DNA sequencing using primer mariner-rev-seq (5′-CAA GCT TGT CAT CGT CAT CC).
