# Supplementary material for: CdiA Effectors Use Modular Receptor-Binding Domains To Recognize Target Bacteria
Source: mBio. 2017 Mar 28;8(2):e00290-17. doi: 10.1128/mBio.00290-17 (PMC5371414; doi:10.1128/mBio.00290-17)
Supplement: FIG S1 [file mbo002173247sf1.pdf]

```
EC93     MHQPPVRFTYRLLSYLVSAIIAGQPLLPAVGAVITPQNGAGMDKAANGVPVVNIATPNGA 60
EC536    MHQPPVRFTYRLLSYLISTIIAGQPLLPAVGAVITPQNGAGMDKAANGVPVVNIATPNGA 60
STECO31  MHQPPVRFPYRLLSYLISTIIAGQPLLPAVGAVITPQNGAGMDKAANGVPVVNIATPNGA 60
         ******** *******:*:*****************************************

EC93     GISHNRFTDYNVGKEGLILNNATGKLNPTQLGGLIQNNPNLKAGGEAKGIINEVTGGKRS 120
EC536    GISHNRFTDYNVGKEGLILNNATGKLNPTQLGGLIQNNPNLKAGGEAKGIINEVTGGNRS 120
STECO31  GISHNRFTDYNVGKEGLILNNATGKLNPTQLGGLIQNNPNLKAGGEAKGIINEVTGGNRS 120
         *********************************************************:**

EC93     LLQGYTEVAGKAANVMVANPYGITCDGCGFINTPHATLTTGKPVMNADGSLQALEVTEGS 180
EC536    LLQGYTEVAGKAANVMVANPYGITCDGCGFINTPHATLTTGRPVMNADGSLQALEVTEGS 180
STECO31  LLQGYTEVAGKAANVMVANPYGITCDGCGFINTPRATLTTGRPVMNADGSLQALEVTEGS 180
         **********************************:******:******************

EC93     ITINGAGLDGTRSDAVSIIARATEVNAALHAKDLTVTAGANRITADGRVSALKGEGNVPK 240
EC536    ITINGAGLDGTRSDAVSIIARATEVNAALHAKDLTVTAGANRITADGRVSALKGEGDVPK 240
STECO31  ITINGAGLDGTRSDAVSIIARATEVNAALHAKDLTVTAGANRVTADGRVSALKGEGDVPK 240
         ******************************************:*************:***

EC93     VAVDTGALGGMYARRIHLTSTESGVGVNLGNLYAREGDIILSSSGKLVLKNSLAGGNTTV 300
EC536    VAVDTGALGGMYARRIHLTSTESGVGVNLGNLYARDGDIILSSAGKLVLKNSLAGGNTTV 300
STECO31  VAVDTGALGGMYARRIHLTSTESGVGVNLGNLYAREGDIILSSSGKLVLKNSLAGGNTTV 300
         ***********************************:*******:****************

EC93     TGTDVSLSGDNKAGGNLSVTGTTGLTLNQSRLVTDKNLVLSSSGQIVQNGGELTAGQNAM 360
EC536    TGTDVSLSGDNKAGGNLSVTGTTGLTLNQPRLVTDKNLVLSSSGQIVQNGGELTAGQNAM 360
STECO31  TGTDVSLSGDNKAGGNLSVTGTTGLTLNQSRLVTDKNLVLSSSGQIVQNGGELTAGQNAM 360
         ***************************** ******************************

EC93     LSAQHLNQTSGTVNAAENVTLTTTDDTTLKGRSVAGKTLTVSSGSLNNGGTLVAGRDATV 420
EC536    LSAQHLNQTSGTVNAAENVTLTTTNDTTLKGRSIAGKTLTVSSGSLNNGGTLVAGRDATV 420
STECO31  LSAQHLNQTSGAVNAAENVTLTTTGGITLKGRSVAGKTLTVSSGSLNNGGTLGAGRDATV 420
         ***********:************.. ******:****************** *******

EC93     KTGTFSNTGTVQGNGLKVTATDLTSTGSIKSGSTLDISARNATLSGDAGAKDRALVTVSG 480
EC536    KTGTFSNTGTVQGNGLKVTATDLTSTGSIKSGSTLDISARNATLSGDAGAKDSARVTVSG 480
STECO31  KTGTFSNTGAVQGNGLKVTATDLTSTGSIKSGSTLDISARNATLSGDAGAKDSARVTVSG 480
         *********:****************************************** * *****

EC93     TLENRGRLVSDDVLTLSATQINNSGTLSGAKELVASADTLTTTEKSVTNSDGNLMLDSAS 540
EC536    TLENRGRLVSDDVLTLSATQINNSGTLSGAKELVASADTLTTTEKSVTNSDGNLMLDSAS 540
STECO31  TLENRGRLVSDDVLTLSATQINNSGTLSGAKELVASADTLTTTEKSVTNSDGNLMLNSAS 540
         ********************************************************:***

EC93     STLAGETSAGGTVSVKGNSLKTTTTAQTQGNSVSVDVQNAQLDGTQAARDILTLNASEKL 600
EC536    STLAGETSAGGTVSVKGNSLKTTTTAQTQGNSVSVDVQNAQLDGTQAARDILTLNASEKL 600
STECO31  STLAGETSAGGTVSVKGNSLKTTTTAQTQGNSVSVDVQNAQLDGTQAARDILTLNASEKL 600
         ************************************************************

EC93     THSGKSSAPSLSLSAPELTSSGVLVGSALNTQSQTLTNSGLLQGKASLTVNTQRLDNQQN 660
EC536    THSGKSSAPSLSLSAPELTSSGVLVGSALNTQSQTLTNSGLLQGEASLTVNTQRLDNQQN 660
STECO31  THSGKSSAPSLSLSAPELTSSGVLVASALNTQSQTLTNSGLLQGEASLTVNTQRLDNQQN 660
         *************************.******************:***************
```

```
EC93    GTLYSAADLTLDIPDIRNSGLITGDNGLMLNAVSLSNPGKIIADTLSVRATTLDGDGLLQ 720
EC536   GTLYSAADLTLDIPDIRNSGLITGDNGLMLNAVSLSNPGKIIADTLSVRATTLDGDGLLQ 720
STECO31 GTLYSAADLTLDIPDIRNSGLITGDNGLTLNTASLSNPGKITADTLNVRATTLDGDGLLQ 720
        **************************** **:.******** ****.*************

EC93    GAGALALAGDTLSLGSNGRWLTAGDLSLRGKTLHTAGTTQGQNLTVQADRWANSGSVQAT 780
EC536   GAGALALAGDTLSQGSHGRWLTADDLSLRGKTLNTAGTTQGQNITVQADRWANSGSVLAT 780
STECO31 GAAALALAGDTLSQGSHGRWLTAGDLSLRGKTLNTAGTTQGQNLTVQADRWANSGSVLAT 780
        **.********** **:******.*********:*********:************* **

EC93    GNLTASATGQLTSTGDIMSQGDTTLNAATTDNRGSLLSAGTLSLDGNSLDNSGTVQGNHV 840
EC536   GNLTASATGQLTSTGDIMSQGDTTLKAATTDNRGSLLSAGTLSLDGNSLDNSGTVQGDHV 840
STECO31 GNLTASATGQLTSTGDIMSQGDTTLNAATTDNRGSLLSAGTLSLDGNSLDNSGTVQGNHV 840
        *************************:*******************************:**

EC93    TIRQNGVTNSGTLTGIAALTLAARMDMASPQPALMNNGGSLL-----------TSGDLTI 889
EC536   TIRQNSVTNSGTLTGIAALTLAARM--VSPQPALMNNGGSLL-----------TSGDLTI 887
STECO31 TLHHRSTDNSGTVTGLSGLTLHSADGLTNSGA-LLSQNSLVLSAGDVTNSGRIQGQNITL 899
        *:::... ****:**::.*** :    ..    *:.:.. :*            . ::*:

EC93    TAGSLANSGAIQAADSLTARLTGELVSTAGSKVTSNGEMALSALNLSNSGQWIAKNLTLK 949
EC536   TAGSLVNSGAIQAADSLTARLTGELVSTAGSKVTSNGEMALSALNLSNSGQWIAKNLTLK 947
STECO31 DASSLTSSGAVQSALDLALTLSGDVIAATGSKITALGDARLTGKVLGNQGLISAKTLEVN 959
         *.**..***:*:* .*:  *:*::::::***:*: *:  *:.  *.*.*   **.* ::

EC93    ANSLTSAGDITGVDALTLTVNQTLNNHASGKLLSAGVLTLKADSVKNDGQLQGNATTITA 1009
EC536   ANSLTSAGDITGVDTLTLTVNQTLNNQANGKLLSAGVLTLKADSVTNDGQLQGNATTITA 1007
STECO31 GDSLSNSGEISGVNSLNVTLSGNL--QQHGKMLTGGALNVNARDISNSGQLQGADNRITA 1017
        .:**:.:*:*:**::*.:*:. .*  :  **:*:.*.*.::* .:.*.*****  . ***

EC93    GQLTNGGHLQGET-LTLAASGGVNNRSGGVLMSRNALNVSTATLSNQGTIQGGGGVSLNA 1068
EC536   GQLTNGGHLQGET-LTLAASGGVNNRFGGVLMSRNALNVSTATLSNQGTIQGGGGVSLNV 1066
STECO31 SSLANSGRVQGESGLTLTLLNALTNQTSGVLLSQNVSALSAPVLTNDGTIQGNGKTTLSA 1077
        ..*:*.*::***: ***:  ..:.*: .***:*:*.  :*: .*:*:*****.* .:*..

EC93    TDRLQNDGKILSGSNLTLTAQVLANTGSGLVQAATLLLDVVNTVNGGRVLATGSADVKGT 1128
EC536   TDRLQNDSKILSGSNLTLTAQVLANTGSGLVQAATLLLDVVNTVNGGRVLATGSADVKGT 1126
STECO31 ATQAHNSGKILSGGELTFTTP--DYSGSGWLQATDLLLNVAKLAGNGTVMAANQATLTGN 1135
        : : :*..*****.:**:*:     :*** :**: ***:*.: ...* *:*:..* :.*.

EC93    TLNNTGTFQGADLLVNYHTFSNSGTLLGTSGLGVKGSSLLQNGTGRLYSAGNLLLDAQDF 1188
EC536   TLNNTGTLQGADLLVNYHTFSNSGTLLGTSGLGVKGSSLLQHGTGRLYSAGNLLLDAQDF 1186
STECO31 SLTNRGLFQAAQLNVNTQTITNSGTLLGNQGLTIKGNNLN-NAGGKVFSGGDMLAEMVSL 1194
        :*.* * :*.*:* ** :*::*******..** :**..*  :. *:::*.*::* :  .:

EC93    SGQGQVVATGDVTLKLIAALTNHGTLAAGKTLSVTSQNAVTNGGVMQGDAMVLGAGEAFT 1248
EC536   SGQGQVVATGDVTLKLIAALTNHGTLAAGKTLSVTSQNAITNGGVMQGDAMVLGAGEAFT 1246
STECO31 SGAGQLVALGNLTLKLTRGLTAQGVIAANKQLSVSSQGDITNGATLQGNGITLNAAGRLT 1254
        ** **:** *::****  .** :*.:**.* ***:**. :***..:**:.:.*.*.  :*

EC93    NNGTLTAGKGNSVFSAQRLFLNAPGSLQAGGDVSLNSRSDITISGFTGTAGSLTMNVAGT 1308
EC536   NNGMLTAGKGNSVFSAQRLFLNAPGSLQAGGDVSLNSRSDITISGFTGTAGSLTMNVAGT 1306
STECO31 NNGQLTAGNGTTALSGSGIAMNASGSLQAGGDVSLTSRGDITLDAFTGTTGSLMLTAAGA 1314
        *** ****:*.:.:*.. : :** ***********.**.***:..****:*** :..**:
```

```
EC93     LLNSALIYAGNNLKLFTDRLHNQHGDILAGNSLWVQKDSSGTANSEIINRSGNIETTRGD 1368
EC536    LLNSALIYAGNNLKLFTDRLHNQHGDILAGNSLWVQKDASGGANTEIINTSGNIETHQGD 1366
STECO31  VINTALLYAGNNLSLFASTIRNHHGDMLAGDSLVMQKDVSGAANAEVINTSGNIETTRGD 1374
         ::*:**:******.**:. ::*:***:***:** :*** ** **:*:** ****** :**

EC93     ITMNTAHLLNSWDAISASHEVI--PGSSHGVI--------SPVPENNRWWGVVR------ 1412
EC536    IVVRTGHLLNQREGFSATTTTRTNPSSIQGMGNALVDIPLSLLPDGS--YGYFTREVENQ 1424
STECO31  ITIRTGHLLNQREGINETKSYIPVENVAVPDGAN--SVSVRVGDLGEDGWGYYVKSWSGT 1432
         *.:.*.****. :.:. :       .                   ..  :*

EC93     -------HDG--VEYLAVYWGKGATVPDEYRIRTGDTETVTVSASGHAARISGGADMHIR 1463
EC536    HGTPCNGHGACNITMDTLYYYAPFADSATQRFLSSQNITTVTGADNPAGRIASGRNLSAE 1484
STECO31  AGG---GF---------DAWAVPTEKGATRKFLTGT-TRVDVGATGGDARISAGNNLLID 1479
                .           :          :: :.    . ..* .  .**:.* ::

EC93     AGRLDNEASFILAGGGMTLSGDTLNNQGWQEGTTGKETVWRLASGSLPKAWFTE------ 1517
EC536    AERLENRASFILANGDIALSGRELSNQSWQTGTENEYLVYRYDPKTFYGSYATGSLDKLP 1544
STECO31  ADKLDNTGSHLLASGFVSLSGSQLNNQSFFGYTQDEYNVYRYYGKLAMIPND-GHL---- 1534
         * :*:* .*.:**.* ::***  *.**.:   * .:  *:*

EC93     ---PWYKVYRQVSPDA--TEASGTSPAGQYRAVISAAGDVSASFATDTGNTTVMPRAGGA 1572
EC536    LLSPEFENNTIRFSLD--GREKDYTPGKTYYSVIQAGGDVKTRFTSSINNGTTTAHAGSV 1602
STECO31  QYGDASADDRVTFTLSGAPEYVTRDTGQALRAVIQAGKNVTAVFSSDISNTSTTSNAGRI 1594
                            .      .    :**.*. :*.: *::. .* :.  .**

EC93     GNTITVPSLNSLTPPTVSQGVSGEALLNESGTGITGPVWNDALPDTLKDIPGALSLSGAS 1632
EC536    SPVVSAPVLNTLSQQTGGDSLTQTALQQYEPVVVGSPQWHDELAGALKNIAGGSPLTGQT 1662
STECO31  TNTLAAPEINTPAEKNISPRMAQLAPDGTEMLTVTAPDWTDTITRLTIG--SGTDL-ASG 1651
           .::.* :*: :  . .  ::  *    .   : .* * * :     .  ..  * .

EC93     --VSSYPLPSGNNGYFVPSTDPDSPYLITVNPKLDGLGKVDSSLFAGLYDLLRMQPGEAP 1690
EC536    GISDDWPLPSGNNGYLVPSTDPDSPYLITVNPKLDGLGQVDSHLFAGLYELLGAKPGQAP 1722
STECO31  IVEGNYPLPSGNNGYFVPSADPDSPYLITVNPKLDGLGKVDSSLFAGLYDLLRMHPGQAP 1711
            ..:*********:***:******************:*** ******:**  :**:**

EC93     RETDPAYTDEKQFLGSSYILDRLGLKPEKDYRFLGDAAFDTRYVSNVILNQTGSRYINGT 1750
EC536    RETAPSYTDEKQFLGSSYFLDRLGLKPEKDYRFLGDAVFDTRYVSNAVLSRTGSRYLNGL 1782
STECO31  RETDPAYTDEKQFPGSSYFLDRLGLKPEKDYRFLGDAAFDTRYVSNYMLNQIGGRYINGV 1771
         *** *:******* ****:*****************.******** :*.: *.**:**

EC93     GSDLAQMKYLMDSAAAQQKALGLTFGVSLTAGQVAQLTRSLLWWESVTINGQTVMVPKLY 1810
EC536    GSDTEQMRYLMDNAARQQKGLGLEFGVALTAEQIAQLDGSILWWESATINGQTVMVPKLY 1842
STECO31  GSDTDQMRYLMDNAARAQKALGLKFGVALTADQVAALDQSILWYKAVTIKGQTVMVPEVY 1831
         ***  **:****.**  **.*** ***:*** *:* *  *:**:::.**:*******::*

EC93     LSPEDITLHNGSVISGNNVQLAGGNITNSGSSINAQNDLLLDRTGSIDNLNAGLINAGGA 1870
EC536    LSPEDITLHNGSVISGNNVQLAGGNITNSGSSINAQNGLSLDSTGYIDNLNAGLISAGGS 1902
STECO31  LSPKDVTLQNGSIISGQNVHLAGGNVTNSGSTLMAQNNLTIDSADSLGNLESGLINAGGA 1891
         ***:*:**:***:***:**:*****:*****:: ***.* :* :. :.**::***.***:

EC93     LNLKAIGDIGNISSVISGKTVSLESATGNISNLTRTEQWAMNN----GYNHFSGTDTGPL 1926
EC536    LDLSAIGDISNISSVISGKTVQLESVSGNISNITRRQQWNAGSDSRYGGVHLSGTDTGPV 1962
STECO31  LGLKAMGDINNISATITGKTVRLESLAGNVNNLTRYSHWQLDAPE--DSLALKHTYTGSI 1949
         *.*.*:***.***:.*:**** *** :**:.*:** .:*  .     .   :. * ** :
```

```
EC93     AAVRATDSLFMGAAGDISITGAAVS----------------------------------- 1951
EC536    ATIKGTDSLSLDAGKNIDITGATVSSGGTLGMSAGNDINIAANLISGSKSQSGFWHTDDN 2022
STECO31  ASVSAMDSLDIRADKNISVTGAEIS----------------------------------- 1974
         *:: . *** : *  :*.:*** :*

EC93     ----------------------------------AGDSVLLAAGNDLNMNAIQAGERRRY 1977
EC536    SASSTTSQGSSISAGGNLAMAAGHNLDVTASSVSAGHSALLSAGNDLSLNAVRESKNSRN 2082
STECO31  ----------------------------------AGDRAALIAGNDLSLNAIDRVSSRRH 2000
                                           **. . * *****.:**:   .  *
EC93     GGSGWYETHAVAPTVTAGNSLMLSAGRDVNSQAAGITAENSMDIRAGRDVNMAAESTGAG 2037
EC536    GRSESHESHAAVSTVTAGDNLLLVAGRDVASQAAGVAAENNVVIRGGRDVNLVAESAGAG 2142
STECO31  ANSESHQRSAGLTTITAGDSVMLSAGRDVSSQGAGIAAEDNITVRAGRDVNLLAEESVTG 2060
         . *  ::  *   *:***:.::* ***** **.**::**:.: :*.*****: **.: :*

EC93     DHDSTFSMKTVHDSVRQQGTDMTSGGDITVTAGRDITSVATAVTAKGDIRVNAGHDIVLG 2097
EC536    DSYTSKKKKEINETVRQQGTEIASGGDTTVNAGRDITAVASSVTATGNISVNAGRDVALT 2202
STECO31  SSSYSKKKTVIDETVRQQGAEIASGGDTTITAGRDITAVASSVTATGNISVNAGRDVALT 2120
         .   : . . :.::*****::::**** *:.******:**::***.*:* ****:*:.*

EC93     TATESDYHYSESGETRNRLLSHQTTRTITEDSVTREKGSLLSGNRVTVNAGNNLTVQGSD 2157
EC536    TATESDYHYLETKKKSGGFLSKKTTHTISEDSASREAGSLLSGNRVTVNAGDNLTVEGSD 2262
STECO31  TATESDYHYLETKKKSGGFLSKKTTHTISENSATREAGALLSGNRVTVNAGDNLTVQGSD 2180
         ********* *: :. . :**::**:**:*:*.:** *:************:****:***

EC93     VVADRDVSLAADNHVDVLAATSTDTSWRFKETKTSGLTGTGGIGFTTGSSKTTHDRREAG 2217
EC536    VVADQDVSLAAGNHVDVLAATSTDTSWRFKETKKSGLMGTGGIGFTIGSSKTTHDRREAG 2322
STECO31  VVADRDVSLAAGNHVDVLAATSTDTSWRFKETKKSGLMGTGGIGFTIGSSKTTHDRREAG 2240
         ****:******.*********************.*** ******** *************

EC93     TTQSQSASTIGSTAGNVSITAGKQAHISGSDVIANRDISITGDSVVVDPGHDRRTVDEKF 2277
EC536    TTQSQSASTIGSTAGNVSITAGKQAHISGSDVIANRDISITGDSVVVDPGHDRRTVDEKF 2382
STECO31  TTQSQSASTIGSTAGNVSITAGKQAHISGSDVIANRDISITGDSVVVDPGHDRRTVDEKF 2300
         ************************************************************

EC93     EQKKSGLTVALSGAVGSAINNAVTMAREAKETSDSRLAALKGTQAVLSGVQAGVNHGLQQ 2337
EC536    EQKKSGLTVALSGTVGSAINNAVTSAQETKESSDSRLKALQATKTALSGVQAGQAATMAS 2442
STECO31  EQKKSGLTVALSGTVGSAINNAVTSAQETKESSDSRLKALQATKTALSGVQAGQAAAMAT 2360
         *************:********** *:*:**:***** **:.*::.*******    :

EC93     QSADPNNGIGVSISLNHQQSKSETKYQHDIVSGSTLSAGNNVSVTATGKNKDHNNSGDML 2397
EC536    ATGDPN-ATGVSLSLTTQKSKSQQHSESDTVSGSTLNAGNNLSVVATGKNRGD-NRGDIV 2500
STECO31  ATGDPN-ATGVSLSLTTQKSKSQQHSESDTVSGSTLNAGNNLSVVATGKNRGD-NRGDIV 2418
          :.*** . ***:**. *:***: : : * ******.****:**.*****:.. * **::

EC93     ITGSQIKSGNDTSLNAQNDILLAAAADTRQTTGKNSSKGGGVGVSFGGGTNGGGLSIFAG 2457
EC536    IAGSQLKVGGNTSLDAANDILLSGAANTQKTTGRNSSSGGGVGVSIGAGGNGAGISVFAG 2560
STECO31  IAGSQLKAGGNTSLDAANDILLSGAANTQKTTGRNSSSGGGVGVSIGAG-KGAGISVFAS 2477
         *:***:* *.:***:* *****:.**:*::***:***.*******:*.* :*.*:*:**.

EC93     INGSEGREKGNGTTWTETTLDAGKNVSLTSGRDTTLSGAQVSGEKVTADVGNNLTISSLQ 2517
EC536    VNAAKGSEKGNGTEWTETTTDSGKTVTINSGRDTVLNGAQVNGNRIIADVGHDLLISSQQ 2620
STECO31  VNAAKGSEKGNGTEWTETTTDSGKTVTINSGRDTVLNGAQVNGNRIIADVGHDLLISSQQ 2537
         :*.::* ****** ***** *:**.*::.*****.*.****.*::: ****::* *** *
```

```
EC93    DSDRYDSRQNRVAAGGSFTFGSMSGSGYASISQDKIKSNYDSVREQSGIYAGKDGFDVTV 2577
EC536   DTSKYDSKQTSVAAGGSFTFGSMTGSGYIAASRDKMKSRFDSVAEQTGMFAGDGGFDITV 2680
STECO31 DTSKYDSKQTSVAAGGSFTFGSMTGSGYIAASRDKMKSRFDSVAEQTGMFAGDGGFDITV 2597
        *:.:***:*. ************:**** : *:**:**.:*** **:*::**..***:**

EC93    GNHTQLNGAVIASTATDDKNSLNTNTLGWSDIHNQADYKASHTGISLSGGSGMSASQMVA 2637
EC536   GRHTQLDGAVIASTATPDKNHLDTGTLGFSDLHNEADYKVSHSGISLSGGGSFGDKFQ-- 2738
STECO31 GRHTQLDGAVIASTATPDKNHLDTGTLGFSDLHNEADYKVSHSGISLSGGGSFGDKFQ-- 2655
        *.****:********* *** *:*.***:**:**:****.**:*******..:. .

EC93    SNAIAGAANALTGMSGSSGHAEGTTSSAISGGNLIIRNKESQKQDIAGLSRDPENANGSI 2697
EC536   ----GNMPGGMISAGGHSGHAEGTTQAAVAEGTITIRDRDNQKQNLANLSRDPAHTNDSI 2794
STECO31 ----GNMPGGMISAGGHSGHAEGTTQAAVAEGTITIRDRDNQKQNPADLSRDPAHANDSI 2711
            ..  ..: . .* ********.:*:: *.: **:::.***: *.***** ::*.**

EC93    APIFDREKEQKRLQEAQVISQISGQMSNIVMTYGETEAMKAARKEH------------PG 2745
EC536   SPIFDKEKEQRRLQTVGLISDIGSQVADIARTQGELNALKAAQDKYG-PVPADATEEQRQ 2853
STECO31 SPIFDKEKEQRRLQTVGLISDIGSQVADIARTQGELNALKAAKEATGETLPANATEKQRQ 2771
        :****:****:*** . :**:*..*:::*. * ** :*:***:.

EC93    MSDAQLRETPEYREVMKGYGTGSTPQMVVQAITGVLGGLNAGNPGQVLAGGLNPAVAQLI 2805
EC536   AYLAKLRDTPEYKKEQEKYGTGSDMQRGIQAATAALQGLVGGNMAGALAGASAPELANII 2913
STECO31 EYLAKLRDTPEYKKEQEKYGTGSEIQLGIQAATAALQGLAGGNLAGALAGASAPELAHLL 2831
           *:**:****::  : *****  *  :** *..* ** .** . .***.  * :*:::

EC93    KQATG--DNREANLMAHAVWGALAAQLGGNNAASGAAGAFSGELAARYIIDNYYGG-RTD 2862
EC536   GHHAGIDDNTAAKAIAHAILGGVTAALQGNSAAAGAIGAGTGEVIASAIAKSLYPGVDPS 2973
STECO31 KSTE---KDPAVNAIAHAILGGAVAAMQGNNVAAGAAGAATGELAARAIAGMLYPGVKQS 2888
               .:  .: :***: *. .* : **..*:** ** :**: *  *    * *   .

EC93    NLSEQERQQISMLATIASGIAGGLVGNSTSAAGTGAQAGRNSVENNAMSGLEGFG-TGFQ 2921
EC536   KLTEDQKQTVSTLATLSAGMAGGIASGDVAGAAAGAGAGKNVVENNALSLVARGCAVAAP 3033
STECO31 DLSEEQKQTISTLATVSAGLAGGLTGNSSASAAVGAQSGKNAVDNNYLSVSEKTELEIAK 2948
        .*:*:::* :* ***:::*:***:.... :.*..** :*:* *:** :*

EC93    SYVQAQEALVNNT---NLTDKNGKVLN----PATPEEIKYASDKLVTGSIPEGQ------ 2968
EC536   CRTKVAEQLLEIGAKAGMAGLAGAAVKDMADRMTSDELE----HLITLQMMGND------ 3083
STECO31 -------QTLKNSK-----------------NPAEREKAQQKYDALLEKDIASDKEVIAAC 2985
                 ::                        :: :     *:  .:  ..

EC93    ---------------------------D----PARGLLISWGAGASVFGGELIAPAVGTV 2997
EC536   ---------------------------EITTKYLSSLHDKYGSGAA------SNPNIGKD 3110
STECO31 GNGNAGSSACASARLKVIASKEGYEDGPYNSKYSQQYADAYGQIVNLLDITSVDVQNQQQ 3045
                                                :*  .

EC93    ------AVIGGTLLGGTTDAVKQFLTLKPGEQYS-TTDTLIAAGEGGLTQGKGVIFS-TF 3049
EC536   LTDAEKVELGGSGSGTGTPPP---SENDPKQQNEKTVDKLNQKQESAIK---------KI 3158
STECO31 VKDAMVSYF-MATLGVDQ---------KTAQGYVETTQGLEIAAAS-M----TPLFGQAV 3090
                :  :  *            .  :    *.: *     .  :           .

EC93    INTMGAYLGSKAK----------GEDPTGPMVGNAIGTAL-GNKAGDKFT---------- 3088
EC536   DNTI----KNALK----------DHDIIGTLK-DMDGKPV-PKENGGYWDH--------- 3193
STECO31 ANKITALVDKANKYPSGIGFKINQPEHLAQLDGYSQKKGISGAHNADVFNKAVVDNGVKI 3150
         *.:     .  *            :  . :      . :   . .. :
```

```
EC93     -KEM---------------------LSRGFGSVTSEVTGTVT------------------ 3108
EC536    MQEMQNTLRGLRNHADTLKNVNNPEAQAAYGRATDAI----------------------- 3230
STECO31  ISETPTGVRGITQVQYEI-----P-TKDAAGNTTGNYKGNGAKPFEKTIYDPKIFTDEKM 3204
          .*                        . . * .*.

EC93     -------------GSVIGTVTDYQIEKLGKG------NKEGAK------ 3132
EC536    -------------NKIESALKGYGI------------------------ 3242
STECO31  LQLGQEAAAIGYSNAIKNGLQAYDAKAGGVTFRVYIDQKTGIVSNFHPK 3253
                      . : . :  *
```

Figure S1. Alignment of representative class I, II and III *E. coli* CdiA effectors. The predicted amino acid sequences of $CdiA^{EC93}$ (AAZ57198.1), $CdiA^{EC536}$ (WP_000554175.1) and $CdiA^{STECO31}$ (WP_001385946.1) were aligned using Clustal Omega at http://www.uniprot.org. Domains and peptide motifs are outlined as determined by the EMBL-EBI InterPro protein sequence analysis site. Red bold-face indicates the secretion signal-sequence; green indicates the TPS transport domain; blue indicates FHA-1 peptide repeats (Pfam: PF05594); orange indicates FHA-2 peptide repeats (PF13332), yellow indicates the pre-toxin-VENN domain (PF04829); and purple indicates the variable CdiA-CT toxins. Within the CdiA-CT region, black bold-face indicates the toxin translocation domains. The receptor-binding regions are shown in black bold-face.
