# Supplementary material for: CdiA Effectors Use Modular Receptor-Binding Domains To Recognize Target Bacteria
Source: mBio. 2017 Mar 28;8(2):e00290-17. doi: 10.1128/mBio.00290-17 (PMC5371414; doi:10.1128/mBio.00290-17)
Supplement: FIG S2 [file mbo002173247sf2.pdf]

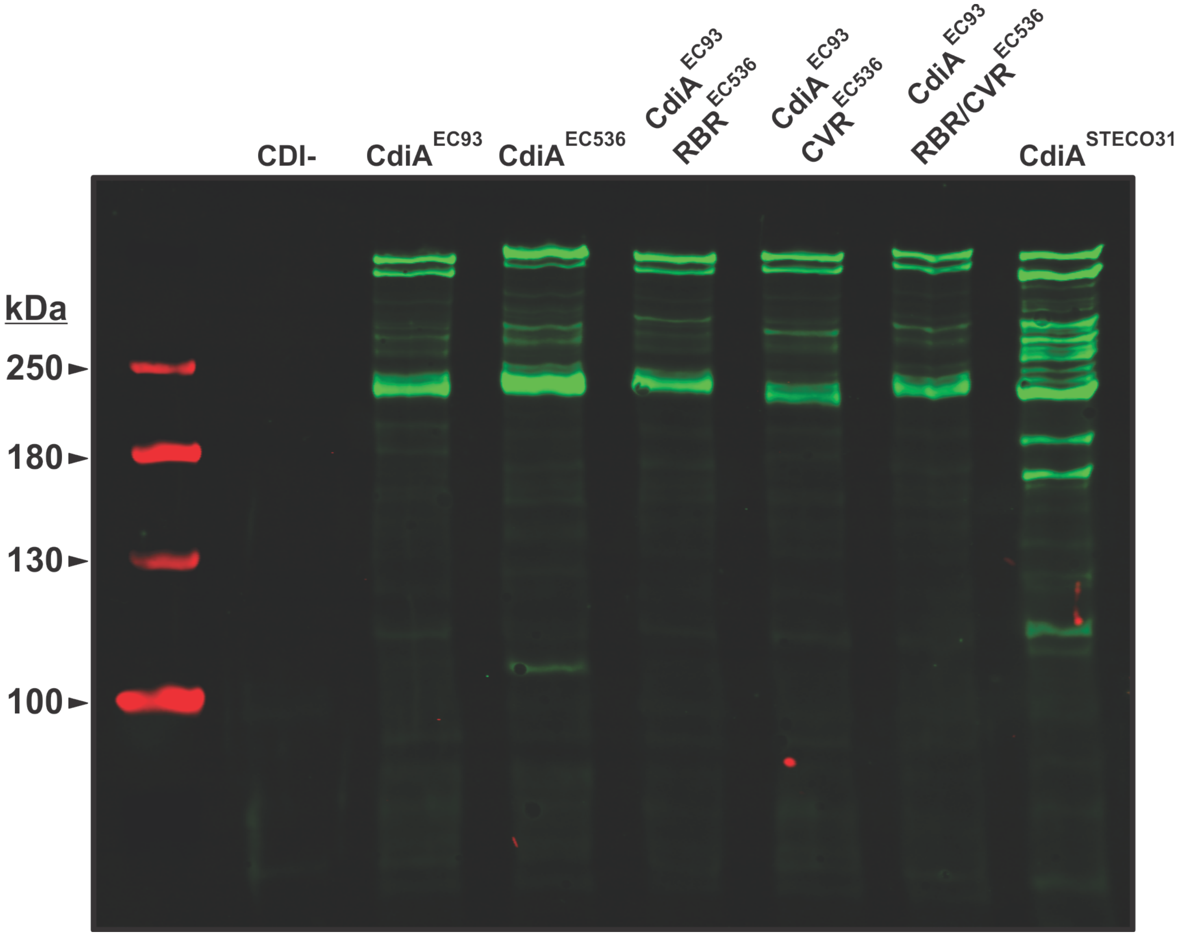

Figure S2. CdiA immunoblot. Total urea-soluble protein from *E. coli* DL4259 cells expressing the indicated CdiA proteins was analyzed by immunoblotting using polyclonal antisera raised against the N-terminal TPS domain of $CdiA^{EC93}$. Predicted molecular masses of CdiA effectors lacking N-terminal signal sequences are ~314 kDa for $CdiA^{EC93}$, ~328 kDa for $CdiA^{EC536}$, and ~320 kDa for $CdiA^{STECO31}$.
