# Supplementary material for: CdiA Effectors Use Modular Receptor-Binding Domains To Recognize Target Bacteria
Source: mBio. 2017 Mar 28;8(2):e00290-17. doi: 10.1128/mBio.00290-17 (PMC5371414; doi:10.1128/mBio.00290-17)
Supplement: FIG S3 [file mbo002173247sf3.pdf]

```
EC93         ---------------------------------------------MHQPPVRFTYRLLSY 15
STECO31_IV   MNRNCYRIIFNKARGMLMVVADIARSGRAGTSLSSRTGYPHRQRICRVTPLAFSLWLASG 60
                                                           :  *: *:  * *

EC93         LVSAIIAGQPLLPAVGAVITPQNGAGMDKAANGVPVVNIATPNGAGISHNRFTDYNVGKE 75
STECO31_IV   MVHSVNAAG-IAADHG--APGHQQPTITQTASGIPQVNIQTPSAGGVSHNTYSQFDVGNK 117
             :* :: *.  :    *     ::   : ::*.*:* *** **...*:*** :::::**::

EC93         GLILNNATGKLNPTQLGGLIQNNPNLKAGGEAKGIINEVTGGKRSLLQGYTEVAGKAANV 135
STECO31_IV   GVILNNAHNNV-QTQLGGMVAGNPWLA-KGEARIILNEVNSRNPSQLNGFVEVAGKKAQV 175
             *:*****  .::  *****:: .** *    ***: *:***.. : * *:*:.***** *:*

EC93         MVANPYGITCDGCGFINTPHATLTTGKPVMNADGSLQALEVTEGSITINGAGLDGTRSDA 195
STECO31_IV   VIANPAGISCDGCGFINANRATLTTGQPQMK-NGSLTGFSVERGEIQITGKGMDASRTDY 234
             ::*** **:*********: :******:* *: :*** .:.* .*.* *.* *:*.:*:*

EC93         VSIIARATEVNAALHAKDLTVTAGANRITADGRVS--ALKGEGNVPKVAVDTGALGGMYA 253
STECO31_IV   TDIIARSVKINAGIWAQDLKVTTGRNNVDIAHGQTEKKAADASSQPQVALDVSSLGGMYA 294
             ..****:.::**.: *:**.**:* *.:      :     . ..  *:**:*..:******

EC93         RRIHLTSTESGVGVNLG-NLYAREGDIILSSSGKLVLKNSLAGGNTTVTG--TDVSLSGD 310
STECO31_IV   GKIRLVGTETGVGVRNAGHIGAQAGAVTLTADGRIENSGSISAKTDVHLATTRELHNSGS 354
              :*:*..**:****. . :: *: * : *::.*::  ..*::. . .   .    ::   **.

EC93         NKAGGNLSVTGTTGLTLNQSRLVTDKNLVLSSSGQIVQNGGELTAGQNAMLSAQHLNQTS 370
STECO31_IV   VYAGQDTQIQS-NGVFTHTGSVASRRNTR--------IQTARLTGGERSLLAAGV--KDD 403
               ** : .: . .*:   : . :.: :*            : ..**.*:.::*:*     : .

EC93         GTVNAAENVTLTTTDDTTLKGRSVAGKTLTVSSGSLNNGGTLVAGRDATVKTGTFSNTGT 430
STECO31_IV   GRLAAAGNLTVSTTGELAAHGQ-------------------VLSGGDMQL---------- 434
             * : ** *:*::**.: : :*:                    :::* *   :

EC93         VQGNGLKVTATDLTSTGSIKSGSTLDISARNATLSGDAGAKDRALVTVSGTLENRGRLVS 490
STECO31_IV   -KGQGLDLSNSRIQGQHTELDATSGNLSTQNVQLSAG-----TLSARTAGHFSNNGGTIN 488
              :*:**.:: : : .   :   ..:: ::*::*. **..         . .:* :.*.*   :.

EC93         DDVLTLSATQINNSG-TLSGAKELVASADTLTTTEKSVTNSD------GNLMLDSASSTL 543
STECO31_IV   ADILQISAQSLSNHRGKLIQT----GTGDFSLNLPGGVDNREGLLAANGAVRLDALSL-- 542
              *:*  :** .:.*    .*   :     .:.*    .    .* *  :        *  : **: *

EC93         AGETSAGGTVSVKGNSLKTTTTAQ--TQGN------SVSVDVQ-----NAQL-------- 582
STECO31_IV   --DNRRGKVQAVQSGSLQVKTTGAVDNQQGSLTASRDVRLNAQALNNDNGLISAAAGTGR 600
               :.   *  . :*:..**:..**.    .*  .       .* ::.*      *.  :

EC93         ----------DGTQAARDILTLNASEKLTHSGKSSAPSLSLS--APELTSSGVLVG-SAL 629
STECO31_IV   IKTQQAVSNTEGRMESAGRLGISAGSLNNHQGTVVSDGLSVTLDGALDNTSGRLLSQKTL 660
                       :*    :  .  *  :.*..   .*.*.   :  .**::   .    .:** *:.  .:*

EC93         N-TQSQTLTNSGLLQGKASLTVNTQR--LDN---QQNGTLYSAADLTLDIPDI-RNSGLI 682
STECO31_IV   SVSGSELVSDDGLIQSGSDMTLDVQDGVLSNRNTKTRGGISSAGTLTVRAGMLNNQQGFI 720
             .  :  *:  :::.**:*.  :.:*::.*    *.*    :  .*  :  **.  **:      :  .:.*:*

EC93         TGDNGLMLNAVSLSNPGKIIADTLSVRATTLDGDGLLQGAGALALAGDTLSLGSNGRWLT 742
STECO31_IV   VGQKDMTLNAGTLDNRQGVLGSQASLQ---ISSGTLMNQKGALKAGTDM--------LLS 769
             .*::.: *** :*.*    ::..   *::    :... *::   ***   .  *             *:

EC93         AGDLSLRGKTLHTAGTTQGQNLTVQADRWANSGSVQATGNLTASATGQLTSTGDIMSQGD 802
STECO31_IV   GGDVSNQEGTL------------------------AAGRDLNAHLNVLENQQGTVVSNGN 805
             .**:*  :   **                          *   :*.*   .    ..  *  ::*:*:

EC93         TTLNAATTDNRGSLLS-AGTLSLDGNSLDNSGTVQGNHVTIRQNGVTNSGTLTGIAALTL 861
STECO31_IV   SRLDVTRFDNQGGRLVAQQSLTLSSTDI-----------INDASGLIQSGASLNLRADTL 854
             :  *:.:   **:*.  *    :*:*....:                    .*:  :**:   .:  *  **
```

```
EC93       AARMDMASPQPALMNNGGSLLTSGDLTITAGSLANSGAIQA-ADSLTARLTGELVSTAGS 920
STECO31_IV SNR---------NSGDRGGVISQGPMTLNAGTLDSTAGVLLSGDAL--SLTAGVVNNTSG 903
           : *           .: *.:::.* :*:.**:* .:..:   .*:*   **. :*..:..

EC93       KVTSNGEMALSALNLSNSGQWIAKNLTLKANSLTSAGDITGVDALTLTVNQTLNNHASGK 980
STECO31_IV QVVANGLLG-----------WNSQALNNQSG------LIQGRGISINTAGQTLDN-RRGT 945
           :*.:** :.           * :: *. ::.       * * .    *..***:*   *.

EC93       LLSAGVLTLKADSVKNDGQLQGNATTITAGQLTNGGHLQGETLTLAASGGVNNRSGGVLM 1040
STECO31_IV LNSLQELTVSTGAMDNRG-----------------GTVGAKTTADLSTTSLDNREGGRLV 988
           * *   **:.:.::.* *                 * : .:* :  :: .::**.** *:

EC93       SRNALNVSTATLSN-QGTIQGGGGVSLNATDRLQ--NDGKILSGSNLTLTAQ-----VLA 1092
STECO31_IV SEGELRLHTGGLQNSHGQIQSVGDMLLNSVRGVVDNVSGLIRSGSAITLNALQFINRHTQ 1048
           *.. *.: *. *.* :* **. *.: **:.  :    .* * *** :**.*

EC93       NTGSGLVQAATLLLDVVNTVNGGRVLATGSADV-KGTT-LNNTGTFQ-GADLLVN--YHT 1147
STECO31_IV NTGQGLEAQTIHITTQDLDNQEGSILADRALTVMADRTLSNNDGVLSSGATLSVSGRQLA 1108
           ***.**   :  :        : * :**  :  *  . *  ** *.:. ** * *.    :

EC93       FSNSGTLLGTSGLGVKGSSLLQNGTGRLYSAGNLLLD-AQDFSGQGQVVATGDVTLKLIA 1206
STECO31_IV FSNRDGVVK-AGQSVSVDAGQLGGDGKLLSLGNMTLKSNTTFSNSGQTIANGNLTLSVNG 1167
           *** . ::  :* .*. .:   .* *:* * **: *.    **..**.:*.*::**.: .

EC93       ALTNHGTLAAGKTLSVTSQNAVT--NGGVMQGDAMVLGAGEAFTNNGTLTAGKGNSV--- 1261
STECO31_IV DVSNTGSLLAGSRLDLNSIRLENTEKGEISAGQ-TWLNVTDTLLNRGLID-GKYTRLQAN 1225
            ::* *:* **. *.:.* .  .  :* :  *:   *.. ::: *.* :  ** . :

EC93       ----FSAQRLFLNAPGSLQAGGDVSLNSRSDITISGFTGT-AGSLTMNVAGTLL---NSA 1313
STECO31_IV TLTNSGTGRIYGDAVGVCAA-------TFNNLEENGVAATLAGRERVDLGVQTLNNRTHS 1278
                .: *:: :* *   *       : .::  .*.:.* **   :::.   *   . :

EC93       LIYAGNNLKLFTDRLHNQHGDILAGNSLWVQKDSSGT-----ANSEIINRSGNIETTRGD 1368
STECO31_IV LIYS--------------AGDMHTGGMLDANGAATGKAGVLNNHSATIEAAGYLVLSAGQ 1324
           ***:                 **: :*. * .:  ::*.       :*  *: :* :  : *:

EC93       ITMNTAHLLNSWDAISASHEVIPGSSHGVISPVPENNRWWGVVR-----HDGVEYLAVYW 1423
STECO31_IV INNVNDHF-------TTERVVVSTEKVTEYQLSGSDKRWSAGEPGVYVDNDSSNSLKKLH 1377
           *.  . *:       ::.: *:  ..    .   .::** .         :*. : *

EC93       -GKGAT-VPDEYRIRTGDTETVTVSASGHAARISGGADMHIRAGRLDNEASFILAGGGMT 1481
STECO31_IV TPEGARDKFTQYDYTR-TVEE-TRVKESDPGKILSGAGMTIVADKLLNDKSQVVAGGLLT 1435
             :**     :*      .*  *   ... .:* .**.* * *.:* *: * ::*** :*

EC93       LSGDTLNNQGWQEGTTGKETVWRLASGSLPKAWFTEPWYKVYRQVSPDAT-EASGTSPAG 1540
STECO31_IV IPSGSVENVSVSG----ERH--------VTDSGTSTYYYRIRKKGKDKQGEKTSQYTPPT 1483
           : ..:::* . .     :.          : .:  :  :*:: :: . .   ::*  :*

EC93       QYRAVISAAGDVSASFATDTGNTTVMPRAGGAGNTITVPSLNSLTPP--TVSQGVSGEAL 1598
STECO31_IV VIQTITLKPGELTSH-----------GQ--VQGSHVTLS---PLKPQGTDVQTGLTGNVD 1527
             :::    *::::                :    *. :*:     *.*    *. *::*:.

EC93       LNESGTG--ITGPVWNDALPDTLKDIPGALSLSGASVSSYPLPSGNNGYFVPSTDPDSPY 1656
STECO31_IV ATVAGTDRIPLRPVVSAGEP--VILLPGQ------Q-FEVSAPQGSIHVAGPDTR----- 1573
            . :**.     ** . . *  :  :**         .  .    *.*.     *.*

EC93       LITVNPKLDGLGKVDSSLFAGLYDLLRMQPGEAPRETDPAYTDEKQFLGSSYILDRLGLK 1716
STECO31_IV ------------LPDSSLFK----TNPAVNVPYLVETDPRFTNQKTWLGSDYMQKAFSQN 1617
                         *****                    **** :*::* :***.*: . :. :

EC93       PEKDYRFLGDAAFDTRYVSNVILNQTGSRYINGTGSDLAQMKYLMDSAAAQQKALGLTFG 1776
STECO31_IV GDNMLKRLGDGFYEQRLIREQVVALTGQRYLDGYSNDEEQFKALMDAGIAFGKQYNLTPG 1677
            ::  : ***. :: * : : ::  **.**::* ..*  *:* ***:. *  *  .** *
```

```
EC93       VSLTAGQVAQLTRSLLWWESVTIN-----GQTVMVPKLYLSPEDITLH-NGSVISGNNVQ 1830
STECO31_IV VALTAEQMALLTGDIVWLVNTTVTLPDGSTQTVQVPQVYARVKPGDVNSAGALIAGRDMV 1737
           *:*** *:* ** .::*  ..*:.      *** **::*   :   ::  *::*:*.::

EC93       -LAGGNITNSGSSINAQNDLLLDRTGSIDNLNAGLINAGGALNLKAIGDIGNISSVISGK 1889
STECO31_IV MKLDGDLFNSGKLAGKQ-----------------------TVQLS-AENIHNQAGTIQGA 1773
              .*:: ***.  . *                       :::*.   :* * :..*.*

EC93       TVSLESATGNISNLTRTEQWAMNNGYNHFSGTDTGPLAAVRATDSLFMGAAGDISITGAA 1949
STECO31_IV NVSL--------------------------------------------TARTDINSTGGL 1789
           .***                                             *  **. **.

EC93       VSAGDSVLLAAGNDLNMNAIQAGERRRYGGSGWYETHAVAP-----TVTAGNSLMLSAGR 2004
STECO31_IV LQATDSLLAMAGRDISLTTTTRTAQRDAGQ-NHFERTRIDSVAGVYVQNDQGRLVLQAGR 1848
           :.* **:*  **.*:.:.:     :*  *  . :*   :       . .  . *:*.***

EC93       DVNSQAAGITAE---NSMDIRAGRDVNMAAESTGAGDHDSTFSM--KTVHDSVRQQGTDM 2059
STECO31_IV DMNLTAATVVNQGKDSLTQLSAGRDMTLSTVTTSAQDNI-TWDKNNRLSQGVTQSTGSTL 1907
           *:*  ** :. :   .  :: ****:.::: :*.* *:  *:.   :  :. .:. *: :

EC93       TSGGDITVTAGRDITSVATAVTAKGDIRVNAGHDIVLGTATES----DYHYSESGETRNR 2115
STECO31_IV AGNGDVTLTAGRDMTSQAASLSAQKGLALMAGHDVTLTGAQNTSSLDEYHKVT---GSSG 1964
           :..**:*:*****:** *::::*: .: : ****:.*  * ::    :**        .

EC93       LLSHQTTRTITEDSVTREKGSLLSGNRVTVNAGNNLTVQGSDVVADRDVSLAADNHVDVL 2175
STECO31_IV MLSKTTTTTHDVSDRRTMTGSELNGDTVSIGAGHNLNVTGSSVAGDNRVSLVAGNNLNIG 2024
           :**: ** *   ..    .** *.*: *::.**:**.* **.*..*. ***.*.*::::

EC93       AATSTDTSWRFKETKTSGLTGTGGIGFTTGSSKTTHDRREAGTTQSQSASTIGSTAGNVS 2235
STECO31_IV MLTESNRETHLKQEKKSGLMSSGGVGFSVGSQSLKV--RDTATDTTQKGSTVGSVHGDVS 2082
             *.:: . ::*: *.*** .:**:**:.**.. .   *::.*  :*..**:**. *:**

EC93       ITAGKQAHISGSDVIANRDISITGDSVVVDPGHDRRTVDEKFEQKKSGLTVALSGAVGSA 2295
STECO31_IV LQAGNRLTVNGSDLIAGRDMALSGKEVSITAATDQHVQTHTVEQKTSGLTLALSGTVGSA 2142
           : **::  :.***:**.**::::*..* :  . *::.  ...***.****:****:****

EC93       INNAVTMAREAKETSDSRLAALKGTQAVLSGVQAGVN---HGLQQQSADPNNGIGVSISL 2352
STECO31_IV LNTTVETVQAAKSAGNSRLEALQGVKAALSGAQAVQAGRLADAQGADAGNNNTVGISLSY 2202
           :*.:*  .: **.:.:*** **:*.:*.***.**       . *  .*. ** :*:*:*

EC93       NHQQSKSETKYQHDIVSGSTLSAGNNVSVTATGKNKDHNNSGDMLITGSQIKSGNDTSLN 2412
STECO31_IV GSQSSKSEQQSEQTVAKGSTLTAGNNLSIQATGSGVK-GVDGDLTIQGSQIKAGNNVLLQ 2261
           . *.**** : :: :..****:****:*: ***.. . . .**: * *****:**:. *:

EC93       AQNDILLAAAADTRQTTGKNSSKGGGVGVSFGGGTNGGGLSIFAGINGSEGREKGNGTTW 2472
STECO31_IV ANRDVNLVSAENTSKLEGKNTSSGGSVGVGVGVGSGGWGISVSASANQGKGSEKGNGTTH 2321
           *:.*: *.:* :* :  ***:*.**.***..* *:.* *:*: *. * .:* *******

EC93       TETTLDAGKNVSLTSGRDTTLSGAQVSGEKVTADVGNNLTISSLQDSDRYDSRQNRVAAG 2532
STECO31_IV TETTVDAGNRLTIISGRDTTLTGAQAGGETVKVDAGRHLTLTSEQDSDRYDSKQQNASAG 2381
           ****:***:.::: *******:***..**.*..*.*.:**::* ********:*:..:**

EC93       GSFTFGSMSGSGYASISQDKIKSNYDSVREQSGIYAGKDGFDVTVGNHTQLNGAVIASTA 2592
STECO31_IV GSFTFGSMSGSASVNLSRDKMHSNYDSVQEQTGIFAGRGGFDVTTGQHTQLNGAVIASTA 2441
           ***********. ..:*:**::******:**:**:**:.*****.*:*************

EC93       TDDKNSLNTNTLGWSDIHNQADYKASHTGISLSGGSGMSASQMVASNAIAGAANALTGMS 2652
STECO31_IV TADKNRLDTGTLGFSDIENRADFKTEHQSAGLSTGGSV------AGNFLGNMANNLLVGA 2495
           * *** *:*.***:***.*:**:*:.* . .** *..:      *.* :.. ** *   :

EC93       GSSGHAEGTTSSAISGGNLIIRNKESQKQDIAGLSRDPENANGSIAPIFDREKEQKRLQE 2712
STECO31_IV NHEGHADSTTQSAVSAGNITIRDTKSQKQDVADLNRDAAHANQTLSPIFDREKEHQRLQQ 2555
           . .***:.**.**:*.**: **:.:*****:*.*.**  :** :::********::***:
```

```
EC93        AQVISQISGQMSNIVMTYGETEAMKAARKEHPGMSDA---------------QLRETPEY 2757
STECO31_IV  AQLIGEIGNQVADIARTEGQIAGEKAKRDPAA-LNQARAELEAAGKPFTEQDVAQRAYNN 2614
            **:*.:*..*:::*. * *:  . ** *.    :.:*                 :.: :

EC93        REVMKGYGTGSTPQMVVQAITGVLGGLNAGNPGQVLAGGLNPAVAQLIKQATGD------ 2811
STECO31_IV  GMAASGFGTGGKYQQAIQAATAAVQGLAGGNLSAALAGGAAPYLAEVVKTMTTDPVTGEV 2674
              . .*:***.. * .:** *..: ** .** . .****  * :*:::*  * *

EC93        NREANLMAHAVWGALAAQLGGNNAASGAAGAFSGELAARYIIDNYYGGRTDNLSEQERQQ 2871
STECO31_IV  NKAANVTAHAVVNAALAVAQGNNALAGAAGAATGEMVG-MIATQMYGKSVSGLSETEKQT 2733
            *: **: **** .*  *   **** :***** :**:..  *  : **  ...*** *:*

EC93        ISMLATIASGIAGGLVGNSTSAAGTGAQAGRNSVENNAMSGLEGFGTGFQSYVQAQEALV 2931
STECO31_IV  LSTLATVAAGLAGGLVGNSGASAVAGAQSGKTTIENNSMSGLVPPRVQQDA------SLA 2787
            :* ***:*:*:******** ::* :***:*:.::***:****    .  ::      :*.

EC93        NNTNLTDKNGKVLNPATPEEIKYASDKLVTGSIPEGQDPARGLLISWGAGASVFGGELIA 2991
STECO31_IV  FDPSQQ---GK-----SAEEISDA-------------IGASHMGPSWGTTYKV---HPIV 2823
             : .     **     : ***. *               *  :  ***:  .*   . *.

EC93        PAVGTVAVIGGTLLGGTTDAVKQFLTLKPGEQYSTTDTLIAAGEGGLTQGKGVIFSTFIN 3051
STECO31_IV  QAGGDVSFIRGYTLNGTIDDN--HISVNQGDIYSIGA----HGGASLGLSFGPYFPGLIN 2877
             * * *:.* *  *.** *    .:::: *: **        * ..*  . *  *  :**

EC93        TMGAYLG-SKAKGEDPTGPMVGNAIGTALGNKAGDKFTKEMLSRGFGSVTSEVTGT-VTG 3109
STECO31_IV  TNNNDYSINGGFGVGSA--------GITMG-KDGVSFTFG-VGPSWGWSATEIKGVDVNG 2927
            * .   . . . * . :        * ::* * * .**   :. .:*  ::*:.*. *.*

EC93        SVIGTVTDYQIEKLGKGNKEGAK 3132
STECO31_IV  TSTNEIYRYDFK----------- 2939
            :  . :  *:::
```

Figure S3. Alignment of representative class I and IV *E. coli* CdiA effectors. The predicted amino acid sequences of CdiA^{EC93} (AAZ57198.1) and CdiA$_2^{STECO31}$ (WP_001081258.1) were aligned using Clustal Omega at http://www.uniprot.org. Domains and peptide motifs are outlined as determined by the EMBL-EBI InterPro protein sequence analysis site. Red bold-face indicates the secretion signal-sequence; green indicates the TPS transport domain; blue indicates FHA-1 peptide repeats (Pfam: PF05594); orange indicates FHA-2 peptide repeats (PF13332), yellow indicates the pre-toxin-VENN domain (PF04829); and purple indicates the variable CdiA-CT toxins. The receptor-binding region of CdiA^{EC93} is shown in black bold-face.
