# Supplementary material for: CdiA Effectors Use Modular Receptor-Binding Domains To Recognize Target Bacteria
Source: mBio. 2017 Mar 28;8(2):e00290-17. doi: 10.1128/mBio.00290-17 (PMC5371414; doi:10.1128/mBio.00290-17)
Supplement: FIG S4 [file mbo002173247sf4.pdf]

```
STECO31-IV MNRNCYRIIFNKARGMLMVVADIARSGRAGTSLSSRTGYPHRQRICRVTPLAFSLWLASG 60
SWW33      MNSLFYRIVFNKARGMLMVVADIARRGRGGNTRRGRC--APAGIIASLSPVCFAIWLAAG 58
           **   ***:**************** **.*.:  .*        *. ::*:.*::***:*

STECO31-IV MVHSVNAAGIAADHGAPGHQQPTITQTASGIPQVNIQTPSAGGVSHNTYSQFDVGNKGVI 120
SWW33      LCSFPAYSAIVADRNAPGNQQATVISTANGLPQVNIQTPGPEGVSRNQYTQFDVDSRGAI 118
           :       :.*.**:.***:** *: .**.*:********.   ***:* *:****..:*.*

STECO31-IV LNNAHNNVQTQLGGMVAGNPWLAKGEARIILNEVNSRNPSQLNGFVEVAGKKAQVVIANP 180
SWW33      LNNSHSNTQTRLAGNITGNPWLAKGEARIILNEVNSRDPSRLNGFIEVAGRRAEVVIANP 178
           ***:*.*.**:*.* ::*******************:**:****:****::*:******

STECO31-IV AGISCDGCGFINANRATLTTGQPQMKNGSLTGFSVERGEIQITGKGMDASRTDYTDIIAR 240
SWW33      SGITCNGCGFINAERTTLAAGKALMDGGRLKGFDVDKGRLSIEGRGLLAEDSDYTALIAR 238
           :**:*:*******:*:**::*:   *..* *.**.*::*.:.* *:*: *.  :*** :***

STECO31-IV SVKINAGIW-AQDLKVTTGRNNVDIAHGQTEKKAADASSQPQVALDVSSLGGMYAGKIRL 299
SWW33      AVNVNARMQVGGELRITTGHNETDLRGNITRIKPGEDAGKPAFALDVSALGGMYAGKIIM 298
           :*::** :   . :*::***:*:.*:   . *. * .: :.:* .*****:********* :

STECO31-IV VGTETGVGVRNAGHIGAQAGAVTLTADGRIENSGSISAKTDVHLATTRELHNSGSVYAGQ 359
SWW33      EGTEKGVGVRNAGELGAMAGTLSLRADGQLVNSGVLAGQGDLSVAAKGDLGNTGSLTAGG 358
            ***.********.:** **:::* ***:: *** ::.: *: :*:. :* *:**: **

STECO31-IV DTQIQSNGVFTHTGSVASRRNTRIQTARLTGGERSLLAAGVKDDGRLAAAGNLTVSTTGE 419
SWW33      DVELTAPGTLSNEGIIRGGQDVRLTAAGIYSSEKSTLLAGAPAATTDRTKGDLVLTSGGE 418
           *.:: : *.::: * : . ::.*: :* : ..*:* * **.       : *:*.::: **

STECO31-IV LAAHGQVLSGGDMQLKGQGLDLSNSRIQGQHTELDATSGNLSTQNVQLSA-GTLSARTA- 477
SWW33      LKARGQNKAAGKIVASGTGVDVSGSRTEAKAVTLTATEREISTAGAVLRGQDTVSLRAAT 478
           * *:**  :.*.:  .* *:*:*.** :.: . * **. ::** .. * . .*:* *:*

STECO31-IV ------------------------------------------GHFSNNGGTIN--------- 488
SWW33      GIDNTAGRVSGGRLTIHSPEITNTGGLLNQEGTQDLLLSQHTLRNNGGSITSAGRIHLKA 538
                                                     : ****:*.

STECO31-IV ------------------------------------------------------------
SWW33      DVLDNRSGTLAAAGRDLSIETGDLDNRQGKVQLSGAGVLSLQTNVLQGQQGALLSSGALR 598

STECO31-IV --------------ADILQISAQSLSNHRGKLIQTGTGDFSLNLPGGVDNREGLLAANGA 534
SWW33      LKAGQADLSGGYTQAGKITAEAGILTNDDAVLSARGQEGMQLTVSGTLSNRNGRAESAGD 658
                         *. :  .*  *:*. . *   *  .:.*.: * :.**:*   : *

STECO31-IV VRLDALSLDNRRGKVQAVQSGSLQVKTTGAVDNQQGSLTASRDVRLNAQALNNDNGLISA 594
SWW33      ITLSSAKLDNSAGTLLAADKGSLSLTTPGEVLNDKGRLIAGRHILLDTAALTSREGLISA 718
           : *.: .***  *.: *.:.***.:.* * * *::* * *.*.: *:: **.. :*****

STECO31-IV AAGTGRIKTQQAVSNTEGRMESAGRLGISAGSLNNHQGTVVSDGLSVTLD-GALDNTSGR 653
SWW33      TGGNMTLNSRQAVINEQGRMEALNRLTLNATGLHNHNGTITGQDVTVGTGVAKLHNTGGN 778
           :.*.  ::::*** * :****: .** :.* .*:**:**:..:.::*   .  . *.**.*.

STECO31-IV LLSQKTLSVSGSELVSDDGLIQSGSDMTLDVQDGVLSNR--------------------- 691
SWW33      IAARDTLALSTGETDNTGGLIQAGKSLVIDTNNRQLSNREGGQILAGALTLSAGNTDNQA 838
           : ::.**::* .*  . .****:*..:.:*.::  ****

STECO31-IV ------------------------------------------------------------
SWW33      GVIASSGDAQLQTADLNSDSGKILSQGDLTLNTAVLRNQHGLLQADGALTLDTHGNGLIS 898

STECO31-IV -NTKTRGGISSAGTLTVRAGMLNNQQGFIVGQKDM-TLNAGTLDNRQGVLGSQASLQISS 750
SWW33      TDSGEQGGIIAGGDLSLRTGRLEAAQGVIQGRNITLDTQGQALDHRGGYLAARESVRLDT 958
            ::  :*** :.* *::*:* *:  **.* *::     :. :**:* * *.:: *:::.:
```

```
STECO31-IV GTLMNQKGALKAGTDMLLSGGD------VSNQEGTLAAGRDLNAHLNVLENQQGTVVSNG 804
SWW33      GETDNSGGLLRAGTSLILDTHDRKLVNQQSGEQGGIVAGDRLTLEVNGLDNHDGVIVSGG 1018
           *   *. * *:***.::*.  *       *.::* :.**  *. .:* *:*::*.:**.*

STECO31-IV NSRLDVTRFDNQGGRLVAQQSLTLSSTDIINDASGLIQSGASL--NLRADTLSNRNSGDR 862
SWW33      DGVMTTGLLDNTQGQLVSASGSLSLTTGEVNNRQGLIQAGKQLLLDTRGQTLVNRDSGEH 1078
           :. : .  :**  *:**: ..    :*. :*: .****:* .*  : *.:** **:**::

STECO31-IV GGVISQGPMTLNAGTLDSTAGVLLSGDALSLTAGVVNNTSGQVVANGLLGWNSQALN-NQ 921
SWW33      GGIRAQGDLSLLSGRLDNHQGIVSAGGQAGLKTVMSDNTAGLITALRGLAITGGQLDNTA 1138
           **: :** ::* :* **.  *:: :*.  .*.: : :**:* :.*   *. ..  *: .

STECO31-IV SGLIQGRGISINTAGQTLDNRR----GTLNSLQELTVSTGAMDNR--------------- 962
SWW33      GSILSGAGLTIDTQGNRLINRDTVSRGGISAAGEAIITTGETDNRSGRIVSDGNAVLHTG 1198
           ..::.* *::*:* *: * **     * :.:  *  ::**  ***

STECO31-IV ------------------------------------------------GGTVGAKTTADLS 975
SWW33      NLKNSLGLIAGNGGLSVNSGETDNTGGRLQSAGDLLINTGTAALVNTGGRIAGDHHTAVT 1258
                                                           ** :...  : ::

STECO31-IV TTSLDNR--------------------EGGRLVSEGELRLHTGGLQNSHGQIQSVGDMLL 1015
SWW33      TSQLTNREGTVQAGERLTLSVGQALDNGKGALLSGGRLALSADRLDNRQGQVIADGDSQL 1318
           *:.* **                    * *:* *.* * :. *:* :**: : **  *

STECO31-IV NSVRGVVDNVSGLIRSGSAITLNALQFINRHTQNTGQGLEAQTIHITTQDLDNQEGSILA 1075
SWW33      N-VQTVLNNESGLVHSAGSLEIVASEINNRHTGQSGKGLEAGKLTLAADILDNAEGAVRG 1377
           * *: *::* ***::*..:: : * :: **** ::*:**** .: :::: *** **:: .

STECO31-IV DRALTVMADRTLSNNDGVLSSGATLSVSGRQLAFSNRDGVVKAGQSVSVDAGQLGGDGKL 1135
SWW33      VSHLTARVTRVLDNLRGLLSSQKTLSVQGQALTVNNREGMLIADDSADISALAVSGDGQI 1437
              **. . *.*.*  *:***  ****.*: *:..**:*:: *.:*..:.*  :.***::

STECO31-IV LSLGNMTLKSNTTFSNSGQTIANGNLTLSVNGDVSNTGSLLAGSRLDLNSIRLENTEKGE 1195
SWW33      LSRDRLTVHVADDFLNTGSVKANGDLTLLTDRRLINDGVIAGQQGLEIRADNLVNTVLGD 1497
           ** ..:*::    * *:*.. ***:*** .:  : * * : . . *::.: .* **  *:

STECO31-IV ISAGQTWLNVTDTLLNRGLIDGKYTRLQANTLTNSGTGRIYGDAVGVCAATFNNLEENGV 1255
SWW33      ITAKETHLNVAGTLENRGLIDGELTHLTTGVLNNTGSGRIFGDHLAVEAGVLNNDRDGDK 1557
           *:* :* ***:.** *******: *:* :..*.*:*:***:** :.* *..:** .:..

STECO31-IV AATLAGRERVDLGVQTLNNRTHSLIYSAGDMHTGGMLDANGAATGKAGVLNNHSATIEAA 1315
SWW33      APVIASRDRLDIAAGTVNNRGHALITSLGNMVFGRHLDNDYRATGRGDVLNNDGAFIEAG 1617
           * .:*.*:*:*:.. *:*** *:** * *:*  *  ** :  ***:..****..* ***.

STECO31-IV GYLVLSAGQINNVNDHFTTERVVVSTEKVTEYQLSGSDKRWSAGEPGVYVDNDSSNSLKK 1375
SWW33      SDAFIGMQTVNNTNRKLETHTVLVEKSQHHEGVLNGSTTRYDWADVDLSKKN--KYGVHT 1675
           .  .:.   :**.* :: *. *:*...:  *  *.** .*:. .: .:  .*  . .::.

STECO31-IV LHTPE-GARDKFTQYDYTRTVEETRVKESDPGKILSGAGMTIVADKLLNDKSQVVAGGLL 1434
SWW33      ARMPDGSENDRFYEYNYTRTVTETQIASTDPGKILAGGNIRFDTARLFNHDSQIVAGGSL 1735
            : *: . .*:* :*:***** **:: .:******:*..: : : :*:*..**:**** *

STECO31-IV TIPSGSVENVSVSGERHVTDSGTSTYYYRIRKKG-----KDKQGEKTSQYTPPTVIQTIT 1489
SWW33      DGHIGTLDNRATQGERVTTDEGWQTRWWPKKKKRPIGGTKTSQGRETDDYRPAPVTETID 1795
               *:::* :..*** .**.* .* ::  :**      * .**.:*.:* *  * :**

STECO31-IV LKPGELTSHGQVQ-----GSHVTLSPLKPQ----GTDVQTGLTGNVDA--TVAGTDRIPL 1538
SWW33      LKSLTWQGHVVVPDKTWRGSERQVSAVNESAEGAGSHSVAGADSRVNISTGQAGMV-STD 1854
           **     .*  *       **.  :* :: .    *:.  :*  ..*:      **

STECO31-IV RPVVSAGEPVILLPGQQFEVSAP---------QGSIHVAGPDTRLPDSSLFKTNPAVNVP 1589
SWW33      IPGVVHDRPLLLPPGHTFSLTLKPETGSGQQITPVIRTVSPDVRLPDNSLFTLHPGTDSH 1914
            * *  ..*::* **: *.::              *:...**.****.***. :*..:
```

```
STECO31-IV YLVETDPRFTNQKTWLGSDYMQKAFSQNGDNMLKRLGDGFYEQRLIREQVVALTGQRYLD 1649
SWW33      YLVETDPRFVNKKQWLSSDYMQNAFTASHDNVHKRLGDGYYEQRLVRDQLIQLTGGRYTG 1974
           *********.*:* **.*****:**: . **: ******:*****:*:*:: *** ** .

STECO31-IV GYSNDEEQFKALMDAGIAFGKQYNLTPGVALTAEQMALLTGDIVWLVNTTVTLPDGSTQT 1709
SWW33      NQSNDEAQYRMLMNNGIAFGKQYQLIPGVALTPEQMALLTSDIVWLVDIPVTLSDGSIQQ 2034
           . **** *:: **: ********:* ****** *******.******:  *** *** *

STECO31-IV VQVPQVYARVKPGDVNSAGALIAGRDMVMKLDGDLFNSGKLAGKQTVQLSAENIHNQAGT 1769
SWW33      VRVPQVYAKLREGDLGGDGALLGGQNVLLSAEQDITGSGNIVGRDVTQLSARTLIN-SGS 2093
           *:******::: **:.. ***:.*:::::. : *: .**::.*::..****..: * :*:

STECO31-IV IQGANVSLTARTDINSTGGLLQATDSLLAMAGRDISLTTTTRTAQRDAGQNHFERTRIDS 1829
SWW33      ISGNRVSLLAGEDILNTGGQILGGKAVSLLAGRNITSETTTRS----DGVN----RWVDR 2145
           *.* .*** *  ** .*** : . .::  :***:*:  ****:     * *      :*

STECO31-IV VAGVYVQNDQGRLVLQAGRDMNLTAATVVNQGKDSLTQLSAGRDMTLSTVTTSAQDNITW 1889
SWW33      RAGIYSEGADGHLTLRALNNITLTGSDIRNAGENGKTSLTAGHDLRLDTVSTVRSQESDW 2205
            **:* :. :*:*.*:* .::.**.: : * *::. *.*:**:*: *.**:*  .::  *

STECO31-IV DKNNRLSQGVTQSTGSTLAGNGDVTLTAGRDMTSQAASLSAQKGLALMAGHDVTLTGAQN 1949
SWW33      GKDNWRREHIQTESGTRIHAAGDLVLSAGRDISATAADVTTDAALTAQAGRDLRLNAGNS 2265
           .*:*   : :  .:*: : . **:.*:****::: **.:::: .*:  **:*: *...:.

STECO31-IV TSSLDEYHKVTGSSGMLSKTTTTTHDVSDRRTMTGSELNGDTVSIGAGHNLNVTGSSVAG 2009
SWW33      VTDLAEH-SKESSRGLLSGHSSERHDEVHTRQAVSTELSGETVHLQSGRDISVSGSNVVS 2324
           .:.* *: .  .* *:**  ::  **  . *  ..:**.*:** : :*:::.*:**.*..

STECO31-IV DNRVSLVAGNNLNIGMLTESNRETHLKQEKKSGLMSSGGVGFSVGSQSLKVRDTATDTTQ 2069
SWW33      SGNLALQAGRGLDITTATESRDETHRREEKKSGLMSSGGIGFTVGKQSLKQSTDSDSRLN 2384
           ...::* **..*:*   ***. *** ::***********:**:**.****    : .  :

STECO31-IV KGSTVGSVHGDVSLQAGNRLTVNGSDLIAGRDMALSGKEVSITAATDQHVQTHTVEQKTS 2129
SWW33      KGSTLGSTDGNVVMTAGGDIKVHGSDVVAKKDISLTGQSVAVTAAENTRTELTKTEQKQS 2444
           ****:**..*:* : **. :.*:***::* :*::*:*:.*::*** : :.:  ..*** *

STECO31-IV GLTLALSGTVGSALNTTVETVQAAKSAGNSRLEALQGVKAALSGAQAVQAGRLADAQGAD 2189
SWW33      GFTLALSGTAGAALNTAVQTAGDAKETDNSRIKALQSIKAGLSGVQGAEAARLAETQGDA 2504
           *:*******.*:****:*:*.  **.:.***::***.:**.***.*..:*.***::**

STECO31-IV AGNNNTVGISLSYGSQSSKSEQQSEQTVAKGSTLTAGNNLSIQATGSGVKGVDGDLTIQG 2249
SWW33      ---GSAFGINLSYGSQSSESETETRQSTAQGSSISAGQNLTVNAGGK--TAGSGNIDVTG 2559
              ..:.**.********:** ::.*:.*:**:::**:**:::* *.  .. .*:: : *

STECO31-IV SQIKAGNNVLLQANRDVNLVSAENTSKLEGKNTSSGGSVGVGVGVGSGGWGISVSASANQ 2309
SWW33      SELQAGGHATLNAQNDIMLTSAQNIQTVDGKNSSKGGSVGVGVTFGSNGAGFNVNASVNR 2619
           *:::**.:. *:*:.*: *.**:* ..::***:*.******** .**.* *:.*.**.*:

STECO31-IV GKGSEKGNGTTHTETTVDAGNRLTIISGRDTTLTGAQAGGETVKVDAGRHLTLTSEQDSD 2369
SWW33      GKGFEKGNSQYATDSTVNAGKTLTLSSGHDTTLKGAQVQGNKVIANVGNNLTLQSEQATD 2679
           *** ****.   *::**:**: **: **:****.***. *:.* .:.*.:*** *** :*

STECO31-IV RYDSKQQNASAGGSFTFGSMSGSASVNLSRDKMHSNYDSVQEQTGIFAGRGGFDVTTGQH 2429
SWW33      NYDSKQTSLSAGGSVGFGN--GSLNISASRDKMHSEYRSVQNQTGIFAGKEGFDITVGKH 2737
           .***** . *****. **.  ** .:. *******:* ***:*******: ***:*.*:*

STECO31-IV TQLNGAVIASTATADKNRLDTGTLGFSDIENRADFKTEHQSAGLSTGGSVAGNFLGNMAN 2489
SWW33      TQLDGAVIVSEGNAEKNRLDTGTLGFKNIENKAEYRVEHQGGSLSTGGGVGDKLISNMGS 2797
           ***:****.* ..*:***********.:***:*:::.***...*****.*..:::.**..

STECO31-IV NLLVGANHEGHADSTTQSAVSAGNITIRDTKSQKQDVADLNRDAAHANQTLSPIFDREKE 2549
SWW33      ALAMGGNKKESSHNTTHAAVSDGKWIIRDTEHQTQDVATLSRDTENAHSALNKIFDKEKE 2857
            * :*.*::  :..**::*** *:  ****: *.**** *.**: :*:.:*. ***:***
```

```
STECO31-IV HQRLQQAQLIGEIGNQVADIARTEGQIAGEKAKRD--------PAALNQARAELEAAGKP 2601
SWW33      QNRVKEQQLLGEIGVQVRDIVRTEATIRATEKAKADFDIRNYKQDDIDKAKAELIAEGKS 2917
           ::*::: **:**** ** **.***. * . :  :            :::*:*** * **

STECO31-IV FTEQDVAQR----AYNNGMAASGFGTGGKYQQAIQAATAAVQGLAGGNLSAALAGGAAPY 2657
SWW33      AGDRDITALLFNKEVEKNLAESGFGTGGKYTRAMQAATAAVQGVMGGDLKAALANGAAPF 2977
             ::*::        ::.:* ********* :*:*********: **:*.****.****:

STECO31-IV LAEVVKTMTTDPVTGEVNKAANVTAHAVVNAALAVAQGNNALAGAAGAATGEMVGMIATQ 2717
SWW33      IANEIKKQIPDEETDA--NLKRTIAHGIANAALALAKGENVAAQATGAMTGEAIGILAEY 3035
           :*: :*.   *  *.   :  .. **.:.*****:*:*:*. * *:** *** :*::*

STECO31-IV MYGKSVSGLSETEKQTLSTLATVAAGLAGGLVGNSGASAVAGAQSGKTTIENNSMSGLVP 2777
SWW33      IYNKQPGELTEREKENVSAWATLASGLAGGLAGGDTQSVANAAQAGKTTVENNYLSSSQS 3095
           :*.*. . *:* **:.:*: **:*:******.*..  *.. .**:****:*** :*.

STECO31-IV PRVQQDASLAFDPSQQGKSAEEISDAIGASHMGPSWGT-----------TYKVHPIVQAG 2826
SWW33      VEFDKELTNC--RKSGGN-CQN---------VIDKWKKVSDEQSAIVDERLESHPLTAVG 3143
            ..::: : .   .. *: .::         :  .* .             : **:. .*

STECO31-IV GDVSFI-------RGYTLNGTIDDNHISVNQGDIYSIGAHGGA--SLGLSFGPYFP--GL 2875
SWW33      WDKEVALGGIDMTERPGWLGSIGADVMTSDEAKAYVQQWNGQDLAKIDMNSPEWMKYALF 3203
            * ..        .     *:*. : :: ::.. *    :*    .:.:.   ::    :

STECO31-IV INTNNNDYSINGGFGVGSAGITMGKDGVSF-----------TFGVGPSWGW--------- 2915
SWW33      VSDPENQVAV-ASLGL--LAKDIGVAAISFMRRNTATATVSASEIGLKWGQGNMKQGMPW 3260
           :.  :*: :: ..:*:   .  :*  .:**           :  :* .**

STECO31-IV SATEIKGVDVNGTSTNEIYRYDFK------------------------------------ 2939
SWW33      EDYVGTTLPANSRLPQNFKTFDYYDEASRVAVSAKSMDTQTMAKLANPNQVYYSIKGNID 3320
           .    . : .*.   :::  :*:

STECO31-IV ------------------------------------------------------------
SWW33      AAAKFEKASLSGVNIDSSMIASKEVRLAVPANTTKTQWTEINRAVEYGKSQGVKVTVTQV 3380


STECO31-IV -
SWW33      K 3381
```

Figure S4. Alignment of representative class IV and V *E. coli* CdiA effectors. The predicted amino acid sequences of CdiA$_{2}^{\mathrm{STECO31}}$ (WP_001081258.1) and CdiA$^{\mathrm{SWW33}}$ (WP_001764992.1) were aligned using Clustal Omega at http://www.uniprot.org. Domains and peptide motifs for CdiA$_{2}^{\mathrm{STECO31}}$ are outlined as determined by EMBL-EBI InterPro, but there are currently no available annotations for CdiA$^{\mathrm{SWW33}}$. Red bold-face indicates the secretion signal-sequence; green indicates the TPS transport domain; blue indicates FHA-1 peptide repeats (Pfam: PF05594); orange indicates FHA-2 peptide repeats (PF13332), yellow indicates the pre-toxin-VENN domain (PF04829); and purple indicates the variable CdiA-CT toxins. Within the CdiA-CT region, black bold-face indicates the toxin translocation domain.
