# Supplementary material for: CdiA Effectors Use Modular Receptor-Binding Domains To Recognize Target Bacteria
Source: mBio. 2017 Mar 28;8(2):e00290-17. doi: 10.1128/mBio.00290-17 (PMC5371414; doi:10.1128/mBio.00290-17)
Supplement: TABLE S1 [file mbo002173247st1.pdf]

Table S1. Predicted class I CdiA proteins encoded by *E. coli* isolates.

CdiA-CT toxin type	NCBI reference ID	*E. coli* isolate	Class I receptor-binding region polymorphisms relative to CdiA-EC93																
			H1395R	K1425E	E1441D	S1448T	H1450Y	G1479S	G1508D	K1521R	P1528T	A1530V	A1539V	R1543H	G1550S	A1572S	V1593G	S1629Y	S1635N
6 - Endonuclease NS_2 (pfam13930)	WP_001075561.1	O26:H11 str. CVM9952		X				X			X								
9 - EC3006 (tRNase)	EKI34460.1	3006			X		X	X		X									X
	WP_065682934.1	1409160003			X			X									X		
	WP_065695502.1	1512290008; 1409150006; 1408270010			X			X									X		
10 - EC93/M605 (membrane pore)	AAZ57198.1	EC93																	
	WP_001501180.1	88.1467		X				X			X				X				
	ERA31492.1	UMEA 4075-1	X		X	X		X	X	X		X				X		X	
	EQZ53807.1	UMEA 3656-1			X		X	X		X									
	WP_065702256.1	1512290008			X			X									X		
	WP_065682929.1	1409150006; 1409160003; 1408270010			X			X									X		
12 - EC869 (tRNase)	WP_001075571.1	O157:H7 str. FRIK944; O157:H7 str. FRIK2533; O157:H7 str. FRIK2455; STEC 757; STEC 690; CFSAN025106; OLC-469; 1303; O26:H11 str. 05-3646; O69:H11 str. 06-3325; NE037; FRIK1997; PA41; FRIK1996; FDA517; FRIK920; TW09109; O26:H11 str. CVM10026; O157:H7 str. EC869; O157:H7 strain FRIK2069		X				X			X				X				
	WP_047091232.1	CFSAN026796		X				X			X				X				
	WP_032277061.1	O118:H16 str. 2009C-4446	X	X				X			X								
14 - Ntox25 (pfam15530)	WP_021575679.1	UMEA 3694-1			X		X	X				X							
	WP_001618261.1	KTE215			X		X	X				X							
	WP_021535355.1	HVH 153	X		X	X		X	X	X			X	X		X			
	WP_001608982.1	KTE194	X		X	X		X	X	X			X	X		X			
	WP_021537513.1	HVH 171			X		X	X				X							
	WP_021549104.1	UMEA 4207-1; KOEGE 43			X		X	X				X							
	WP_001518495.1	KTE15			X		X	X				X							
	WP_021568213.1	UMEA 3268-1			X		X	X				X							
	WP_021568012.1	UMEA 3264-1			X		X	X				X							
	WP_033561291.1	UPEC-184	X		X	X		X	X	X			X	X		X			
	WP_021512687.1	UMEA 3955-1; HVH 228; HVH 4			X		X	X				X							
	WP_023278102.1	UMEA 3693-1			X		X	X				X							
	WP_021532821.1	UPEC-14; HVH 143; HVH 138			X		X	X				X							
	WP_001567070.1	KTE93			X		X	X				X							
	WP_001535250.1	KTE189			X		X	X				X							
	WP_021515481.1	HVH 212			X		X	X				X							
	WP_001612021.1	KTE183			X		X	X				X							
	WP_060588708.1	50870281			X		X	X				X							
	WP_021554696.1	UMEA 3087-1			X		X	X				X							
	WP_021575523.1	UPEC_003; UMEA 3687-1			X		X	X				X							
	WP_021540215.1	HVH 190			X		X	X				X							
	WP_061892950.1	tEC; EC93																	
	WP_064237070.1	UPEC_007			X		X	X				X							
