# Supplementary material for: CdiA Effectors Use Modular Receptor-Binding Domains To Recognize Target Bacteria
Source: mBio. 2017 Mar 28;8(2):e00290-17. doi: 10.1128/mBio.00290-17 (PMC5371414; doi:10.1128/mBio.00290-17)
Supplement: TABLE S2 [file mbo002173247st2.pdf]

Table S2. Predicted class II CdiA proteins encoded by *E. coli* isolates.

CdiA-CT toxin type	NCBI reference ID	*E. coli* isolate	Class II receptor-binding region polymorphisms relative to CdiA-EC536								
			Y1416F	A1497A	D1527G	P1528Q	G1561D	T1567M	P1609A	G1620E	S1622C
1 - DUF769 (pfam05590)	EOU64800.1	KTE19									
	WP_021572694.1	UMEA 3585-1									
2 - 97.0246 (unknown)	EIG93024.1	97.0246				X					
	WP_057728513.1	ECO5_109				X					
	WP_057711373.1	STEC 627; STEC 299; ECO5_188; ECO_097; ECO_196; ECO5_174; ECO_086; ECO5_060				X					
3 - EC1738 (unknown)	WP_047620894.1	CVM N33806PS; CVM N33804PS						X			
	WP_033557998.1	UPEC-95; UPEC-150									
	WP_044869828.1	OLC-733									
	WP_053270965.1	1.EC2992.1 (chicken fecal)									
	WP_021581262.1	UMEA 4076-1									
	ELH39110.1	KTE183									
	CAJ87528.1	A0 34/86									
5 - NC101 (tRNase)	WP_021518057.1	HVH 28									
	WP_023278025.1	UMEA 3290-1									
	WP_021573235.1	UMEA 3617-1									
	WP_021549463.1	KOEGE 58									
	WP_001531945.1	MB3298; swine69; B48; 3019; UPEC-228; UMEA 3113-1; HVN 185; HVH 146; HVH 141; HVH 17; KTE178									
	WP_047085063.1	CFSAN026836; CFSAN026835									
	WP_046122762.1	TW18703; TW18710									
	WP_042021164.1	UPEC-112									
	WP_000554167.1	1512290008; 1409150006; 1408270010; NGF4; NGF2; NGF3; NGF1; UCD-JA19; M17 - 1; UPEC-28; KTE207; KTE16; KTE133; NC101									
	WP_021571728.1	UMEA 3341-1									
	WP_021552902.1	932_ECOL 221_58329_1013568; UPEC-91; UMEA 3217-1; UMEA 3097-1; UMEA 3022-1									
6 - Endonuclease NS_2 (pfam13930)	AJF58502.1	1303						X			X
	EQS75540.1	HVH 162									
7 - O32:H37 (unknown)	KIH04857.1	CVM N33825PS									
	EIF16908.1	O32:H37 str. P4									
10 - EC93/M605 (membrane pore)	WP_047629948.1	CVM N33687PS					X				
	WP_001547418.1	KTE230									
	WP_021524826.1	HVH 74									
	WP_001533898.1	KTE188									
	WP_001564262.1	KTE76		X							
	WP_047649601.1	CVM N36401PS		X							
	WP_033561912.1	K71; CVM N33591PS; UPEC-187		X							
	WP_047657653.1	CVM N36404PS		X							
	WP_046644777.1	avian2									
	WP_058061197.1	TTU2014-120BME						X			
	WP_058061262.1	TTU2014-121AME						X			
	ERA04464.1	UMEA 3805-1									
11 - Ntox28 (pfam15605)	WP_000554175.1	536									
	WP_000554174.1	K-15KW01; ECONIH2; EC75; GN02183; GN03545; 696_ECOL 193_40484_590770; UPEC-60; UPEC-156; UMEA 3426-1; HVH 100; KTE8; MS 60-1; F11									
	ETF18666.1	HVH 23									
	ESK12185.1	UMEA 3290-1									
	EQO02198.1	HVH 28									
	KKA62274.1	9.1649									
	KDY17189.1	2-316-03_S4_C3								X	
	EQQ80302.1	HVH 112									
	ELF79209.1	KTE43								X	
	ELD47760.1	KTE224								X	
	KEJ66099.1	3-020-07_S4_C1								X	
	EQP16128.1	HVH 61									
	EQX13835.1	UMEA 3160-1								X	
	EQV28412.1	KOEGE 30									
	EQZ29384.1	UMEA 3585-1									
	KSW91104.1	K71-77								X	
	EQW66621.1	UMEA 3088-1					X			X	
	WP_045171861.1	UPEC-111									
	EQP63393.1	HVH 78									
	EOX21599.1	KTE185			X				X		
	ERA29139.1	UMEA 3955-1									
	WP_033545329.1	UPEC-38									
	ESK23705.1	UMEA 3693-1									
	EOV05042.1	KTE195									
	ELC78292.1	KTE189									
	ELD03531.1	KTE201									
	EQR32984.1	HVH 121									
	WP_000554170.1	UM141; GN02260; GN02005; GN02172; GN02148; GN02254; LSPQ A134697; 725_ECOL 515_22705_334449; BIDMC114; blood-08-0493; UPEC-169; blood-08-0654; UPEC-129; RS218; SCB12; UPEC-76; UMEA 3834-1; UMEA 3298-1; KTE55; UTI89; UM146									
	WP_020231832.1	TOP382-1								X	
	WP_042043595.1	blood-09-0751									
	WP_033549588.1	blood-10-0687									
	WP_021512800.1	HVH 4									
	ELC94224.1	KTE191									
	WP_033550614.1	UPEC-120									
	WP_021529048.1	HVH 111									
	EGJ05980.1	D9									
	WP_061089048.1	B150; B16; B133; B75; B77; B94									
	WP_059319605.1	GN04124								X	
	WP_059340580.1	GN02099									
	WP_021563313.1	UMEA 3193-1									
	WP_059337576.1	GN02165									
13 - Zn-dependent DNase (cd13444)	WP_001075566.1	TA271	X								
	WP_021535428.1	HVH 153									
	WP_047651975.1	CVM N36400PS									
	KIO86048.1	97.0264									
	WP_061355056.1	RiKo 2308/09;RiKo 2340/09; G76									
	WP_047651975.1	CVM N36400PS									
	WP_061351111.1	G222									
14 - Ntox25 (pfam15530)	WP_001560878.1	KTE72									
	WP_021567981.1	UMEA 3264-1									
	WP_021568211.1	UMEA 3268-1									
	WP_021561478.1	UMEA 3172-1									
	WP_021534665.1	HVH 149									
	WP_021573859.1	UMEA 3656-1									
	WP_021511806.1	HVH 2									
	WP_033559456.1	blood-10-1308; blood-10-1310									
	WP_033553046.1	UPEC-195									
	WP_032284995.1	O119:H4 str. 03-3458									
	WP_021581317.1	UMEA 4207-1									
	WP_000554180.1	TA206									
	WP_039025707.1	EC7; EC5									
	WP_020245261.1	TOP2662-1									
