# Supplementary material for: CdiA Effectors Use Modular Receptor-Binding Domains To Recognize Target Bacteria
Source: mBio. 2017 Mar 28;8(2):e00290-17. doi: 10.1128/mBio.00290-17 (PMC5371414; doi:10.1128/mBio.00290-17)
Supplement: TABLE S3 [file mbo002173247st3.pdf]

Table S3. Predicted class III CdiA proteins encoded by *E. coli* isolates.

CdiA-CT toxin type	NCBI reference ID	*E. coli* isolate	Receptor-binding region polymorphisms relative to CdiA-STECO31													
			I1396V	G1415R	D1438E	G1462V	D1467T	D1481N	G1494E	A1576V	R1614G	A1616T	M1625A	M1625T	A1649V	E1654V
3 - EC1738 (unknown)	WP_001075572.1	9.0111			X				X		X		X		X	
	WP_041124063.1	1303			X				X		X		X		X	
	WP_000554178.1	O121:H19 str. MT#2			X				X		X		X		X	
4 - Bacterial EndoU (pfam14436)	WP_032280889.1	O121:H19 str. 2009C-4050	X		X						X			X		
	WP_001428356.1	CFSAN026796; CVM N36099PS; DEC8D	X		X						X			X		
	WP_001385946.1	STEC_O31														
	WP_001562306.1	KTE75									X			X		
	WP_050877941.1	CFSAN026786								X	X	X				
6 - Endonuclease NS_2 (pfam13930)	WP_048956774.1	1280_ECOL 2362_42339_494385			X				X		X		X			X
	WP_001680718.1	TTU2014-121AME; TTU2014-120BME; TTU2014-148AME; HVH 98; HVH 126; HVH 42; KTE180			X				X		X			X		X
	WP_032215267.1	O121:H7 str.2009C-3299			X				X		X			X		
	EQP98458.1	HVH 89				X	X		X		X		X			
	WP_052870112.1	A192PP				X	X		X		X		X			
	ERF50383.1	UMEA 3652-1				X	X		X		X		X			
8 - DUF4258 (pfam14076)	WP_001442268.1	NCCP15647			X		X				X					
	WP_047086875.1	CFSAN026806; CFSAN026802			X		X				X					
	WP_052904243.1	CFSAN026807; CFSAN026808									X					
	WP_044686884.1	FHI89									X					
10 - EC93/M605 (membrane pore)	KDV64783.1	O128:H2 str. 2011C-3317			X				X		X		X			
	WP_050864234.1	CFSAN026784; CFSAN026777								X	X	X				
	WP_050867619.1	CFSAN026781								X	X	X				
	WP_060612756.1	2011C-3198; NCCP 15655; NCCP 15656														
	WP_047091714.1	CFSAN026773														
	WP_052892073.1	CFSAN026782														
	WP_048264129.1	OLC-816; 94-3024 (plasmid)			X		X				X					
	WP_053294085.1	cow fecal		X							X					
	WP_019842636.1	HM26			X			X	X		X			X		
	WP_001499179.1	CFSAN026774; 0.1288			X				X		X			X		X
	WP_032209771.1	O174:H21 str. 03-3269			X				X		X			X		X
	EFE64162.1	B088			X				X		X			X		
	WP_053265048.1	magpie fecal; magpie fecal			X				X		X			X		
	WP_046201737.1	CI5 (plasmid)			X				X		X			X		
	WP_062888789.1	STEC 3098			X				X		X			X		X
