# Supplementary material for: CdiA Effectors Use Modular Receptor-Binding Domains To Recognize Target Bacteria
Source: mBio. 2017 Mar 28;8(2):e00290-17. doi: 10.1128/mBio.00290-17 (PMC5371414; doi:10.1128/mBio.00290-17)
Supplement: TABLE S4 [file mbo002173247st4.pdf]

Table S4. Predicted class IV CdiA proteins encoded by *E. coli* isolates.

CdiA-CT toxin type	NCBI reference ID	*E. coli* isolate
1 - DUF769 (pfam05590)	WP_001081262.1	CFSAN025126; CFSAN025133; CFSAN025119; CFSAN025118; CFSAN025115; CFSAN025122; CFSAN025109; CFSAN025095; CFSAN025124; CFSAN025132; DEC9E; DEC9A
	WP_021519220.1	HVH 38
	WP_049592744.1	KIC-2
	WP_054575869.1	UCD-JA30
	WP_001604133.1	KTE146
	WP_042972592.1	CVM N33561PS
	WP_001081261.1	B185
	WP_040090615.1	CVM N38663PS; CVM N34228PS
	WP_061316050.1	CFSAN025099
	WP_065106288.1	IMT26197
3 - EC1738 (unknown)	WP_001081260.1	3.3884
	WP_047091398.1	CFSAN026773
	WP_047091209.1	CFSAN026796
	WP_061351115.1	G222
	WP_052920175.1	STEC 3031; CFSAN026806 (2)
	WP_052892011.1	CFSAN026782
	WP_001081264.1	28RC1; O157:H7 str. 2011EL-2111; O157:H7 str. 2011EL-2109; O157:H7 str. 2011EL-2114; O157:H7 str. 2011EL-2112; O157:H7 str. 2011EL-2113; O157:H7 str. 2011EL-2105; O157:H7 str. 2011EL-2108; O157:H7 str. 2011EL-2103; O157:H7 str. 2011EL-2107; O157:H7 str. 2011EL-2104; 5.2239; PA10; EC1738
	WP_001081259.1	NCCP15647
	WP_044372903.1	OLC-683
	WP_053913379.1	644-PT8; 180-PT54; STEC 2770; PARC448; STEC 623; STEC 2820; STEC 2821; STEC 2257
	WP_032272778.1	O123:H11 str. 2009C-3307; O111:NM str. 2010C-4735
	WP_021497347.1	22593; OLC-1547; O157:H7 str. F7377; O157:H7 str. F7350; B86; B85; B84; B40-1; B106; B105; B103; B26-1; B107; B102; 95.0943
	WP_032188571.1	2-460-02_S3_C3; 2-460-02_S3_C2; 2-460-02_S3_C1
	EKW33081.1	95.0943
	WP_053264744.1	1.EC2996.1 (magpie fecal, 2)
	WP_065225452.1	GN1221
	WP_065222260.1	GN1222
6 - Endonuclease NS_2 (pfam13930)	EHW22223.1	DEC8D
	WP_059331390.1	GN02350
7 - O32:H37 (unknown)	EHP66680.1	4_1_47FAA
9 - EC3006 (tRNase)	ENE16675.1	P0304799.3
10 - EC93/M605 (membrane pore)	WP_049590174.1	73-89
	WP_024226415.1	C283_09 C283_09_77
	WP_040078269.1	CVM N36099PS
	WP_033811107.1	(human fecal)
	WP_001081251.1	3006
	WP_053295534.1	1.EC2923.1 (swine fecal)
	WP_033801982.1	(human fecal)
	WP_047081959.1	CFSAN026809
	WP_032306931.1	ESC369; STEC 2826; H1.94; CFSAN026785; O91:H21 str. 2009C-3740; O91:H21 str. 2009C-4646
	WP_001081255.1	H2.185; O91:H21 str. B2F1; STEC_B2F1; 96.0497
	WP_047090654.1	87-1714; CFSAN026775; CFSAN026786; CFSAN026810
	WP_032326960.1	O79:H7 str. 06-3501
	WP_052892274.1	CFSAN026803
	WP_054632408.1	PARC444
	WP_001081254.1	B799
	WP_021552436.1	KOEGE 131
	WP_062871665.1	STEC 2236
	WP_062881271.1	STEC 200
	WP_064055993.1	2011C-3911
	WP_064237064.1	UPEC_007
	WP_061329421.1	CFSAN025125; CFSAN025121; CFSAN025123
	WP_050443477.1	BIDMC 83
	WP_029400086.1	435
	ELE47736.1	KTE75
	WP_044187772.1	48
	WP_062871861.1	STEC 1506
	WP_063502791.1	sheep24
	WP_021531610.1	UMEA 3230-1; UMEA 3221-1; KOEGE 56; HVH 125
	WP_021573385.1	UMEA 3632-1
	WP_063121087.1	swine70
	WP_054410865.1	E. albertii 24
	CTV15700.1	102606_aEPEC
	WP_042968498.1	sheep feces
	WP_050876293.1	CFSAN026776
	WP_044807758.1	FHI92
	WP_054627714.1	STEC 2953; 09-0525
	WP_044809568.1	STEC 1363; FHI71
	WP_033884924.1	O128:H2 str. 2011C-3317
	WP_062876712.1	STEC 2074
	EIH23276.1	1.2264
	WP_032192354.1	2-474-04_S4_C1
	WP_032186098.1	2-474-04_S4_C2; 2-474-04_S4_C3
	WP_059337603.1	GN02165
	WP_050864087.1	STEC 1375; CFSAN026784; CFSAN026795; CFSAN026777
	WP_028985513.1	STEC O174:H2 str. 0204446
	WP_044808459.1	FHI59
	WP_052904221.1	CFSAN026807; CFSAN026808
	WP_047082158.1	CFSAN026787
	WP_047642988.1	P.231
	WP_033811935.1	ESC173; ESC256; CFSAN026793; minced meat
	WP_050866939.1	CFSAN026794
	WP_047661926.1	CVM N38733PS
	WP_062865821.1	STEC 2591
	WP_062850925.1	STEC 2064; STEC 886
	WP_062863367.1	STEC 645; STEC 2788; STEC 3106; STEC 29
	WP_062859364.1	STEC 2363; STEC 2174; STEC 1188, STEC 2962
	WP_062877361.1	STEC 196; STEC 329
	WP_045148672.1	FHI30
	WP_001081256.1/ EGI12780.1	M605
	WP_040100933.1	CVM N33825PS
	WP_044804370.1	FHI28
	WP_001420113.1	MP021561.3
14 - Ntox25 (pfam15530)	WP_052920846.1	CFSAN026797
	WP_001081258.1	CFSAN026781; STEC_O31; cow fecal
	WP_053289062.1	sheep15; sheep13; beef cow fecal
	WP_039005204.1	EC4
	WP_044697313.1	FHI98
	WP_047627746.1	CVM N36834PS
	WP_047664262.1	CVM N41556PS
	WP_047660116.1	CVM N41498PS
	WP_063069653.1	sheep19; sheep21; sheep22; sheep16; sheep10, sheep18
